# Immunotheranostic microbubbles (iMBs) - a modular platform for dendritic cell vaccine delivery applied to breast cancer immunotherapy

**DOI:** 10.1186/s13046-022-02501-3

**Published:** 2022-10-12

**Authors:** Natacha Jugniot, Jeremy J. Dahl, Ramasamy Paulmurugan

**Affiliations:** 1 Department of Radiology, Molecular Imaging Program at Stanford, Canary Center for Cancer Early Detection, Stanford University, Palo Alto, CA USA; 2 Molecular Imaging Program at Stanford (MIPS), Canary Center for Cancer Early Detection at Stanford, Stanford University School of Medicine, 3155 Porter Drive, Palo Alto, CA 94304 USA

**Keywords:** Immunotherapy, Oncology, Microbubbles, Dendritic cell vaccine, Breast Cancer, Molecular imaging, Ultrasound (US)

## Abstract

**Background:**

Therapeutic strategies engaging the immune system against malignant cells have revolutionized the field of oncology. Proficiency of dendritic cells (DCs) for antigen presentation and immune response has spurred interest on DC-based vaccines for anti-cancer therapy. However, despite favorable safety profiles in patients, current DC-vaccines have not yet presented significant outcome due to technical barriers in active DC delivery, tumor progression, and immune dysfunction. To maximize the therapeutic response, we present here a unique cell-free DC-based vaccine capable of lymphoid organ targeting and eliciting T-cell-mediated anti-tumor effect.

**Methods:**

We developed this novel immunotheranostic platform using plasma membranes derived from activated DCs incorporated into ultrasound contrast microbubbles (MBs), thereby offering real-time visualization of MBs’ trafficking and homing in vivo. Human PBMC-derived DCs were cultured ex vivo for controlled maturation and activation using cell membrane antigens from breast cancer cells. Following DC membrane isolation, immunotheranostic microbubbles, called DC-iMBs, were formed for triple negative breast cancer treatment in a mouse model harboring a human reconstituted immune system.

**Results:**

Our results demonstrated that DC-iMBs can accumulate in lymphoid organs and induce anti-tumor immune response, which significantly reduced tumor growth via apoptosis while increasing survival length of the treated animals. The phenotypic changes in immune cell populations upon DC-iMBs delivery further confirmed the T-cell-mediated anti-tumor effect.

**Conclusion:**

These early findings strongly support the potential of DC-iMBs as a novel immunotherapeutic cell-free vaccine for anti-cancer therapy.

**Supplementary Information:**

The online version contains supplementary material available at 10.1186/s13046-022-02501-3.

## Background

Immunotherapies have transformed anti-cancer treatment with incomparable advantages over traditional chemotherapies and surgery [1]. Among them, vaccination aims to set off a patient’s own immune response against cancer cells through enhanced tumor antigen presentation to immune cells [2]. Vaccines’ good safety profile constitutes a precious asset compared to adoptive T-cell therapy (ATC), immune checkpoint inhibitors (ICIs), and antibody-drug conjugates, where cytokine release syndrome and off-target effects are commonly associated with mild to life threatening toxicities [3].

DCs are critical antigen-presenting cells (APCs) for potent initiation of adaptive immune response. They sensitize naive CD8^+^ cytotoxic T lymphocytes (CTLs) and CD4^+^ T-cells through the interaction of major histocompatibility complexes (MHC) I-T-cell receptor (TCR) and MHC II-TCR, respectively [4]. Nevertheless, depending on their maturation level, functional state, and subtype, DCs can prompt T-cell-mediated immune tolerance [5]. Of note, studies have shown that DC’s maturation level positively impacts overall prognosis [6, 7]. Additionally, increased number of tumor-infiltrating DCs correlates with improved survival rates in various malignancies [8, 9]. Thus, DC vaccines are the focus of many pre-clinical studies and have been tested in more than 480 clinical trials [10–12]. Current DC vaccine preparation involves the ex vivo manipulation of DCs generated from autologous CD14^+^ monocytes or CD34^+^ hematopoietic precursor cells from a patient’s blood. After quality check, active DCs are re-infused into the same patient at a site facilitating their homing toward the nearest lymphoid tissue for T-cell priming. Maturation and activation processes can be highly controlled compared to potentiation of DCs in vivo [13]. Sporadic successes have yet escalated the need for alternative DC vaccine delivery platforms [14–16]. Many mechanisms have been proposed for this failure and are mainly a combination of the patient’s immune system state and tumor’s complex molecular landscape promoting immune escape [15]. Thus, next-generation DC vaccines are urgently needed to overcome the molecular lockers impeding T-cell–driven therapies.

Microbubbles (MBs) are gas-filled microparticles (1–6 μm in diameter) predominantly synthesized from a combination of lipids [17]. Because of their high compressibility and low density, MBs are highly echogenic. Due to their size, MBs stay confined in the vasculature and are used in clinical ultrasound (US) imaging to monitor blood flow and vascular density. Recently, MBs have also been applied to tumor microvasculature imaging in patients using molecularly targeted MBs [18]. In addition to imaging, MBs can be used as drug delivery enablers through targeted sonoporation (i.e.*,* transient pore formation), or directly as a therapeutic for cancer treatment through antivascular effects [19, 20]. Furthermore, clinical and pre-clinical studies have demonstrated a specific accumulation of MBs in the spleen, the lymph nodes, and the liver as part of their clearance process [21, 22]. MBs are most likely captured by splenic macrophages, mononuclear phagocytes, and by Kupffer cells for phagocytosis. Pulmonary macrophages may also contribute to some MB entrapment during bubble gas core exhalation [23]. Thus, linking the inherent lymphoid organ accumulation of MBs with in situ immune cell activation mediated by DCs could highly potentiate anti-cancer immunotherapies.

To maximize imaging contrast and therapeutic efficacy, MB size and surface properties must be tightly controlled. Microfluidics is a versatile platform for producing uniform MBs by the creation of local gas/liquid interfacial instabilities to “pinch off” small volumes of gases in MBs. We recently developed a microfluidic platform using a pressure-based disruption and reconstitution process based on the inherent self-assembly property of lipids in aqueous solutions to generate a variety of nano- and microparticles [24, 25]. Here, we synthesized immunotheranostic MBs by enriching the lipid phase with DC plasma membrane fragments pulsed against triple negative breast cancer (TNBC) antigens, that we called “DC-iMBs”. Details on MB generation are presented in Supplementary Scheme 1.

**Scheme 1:**
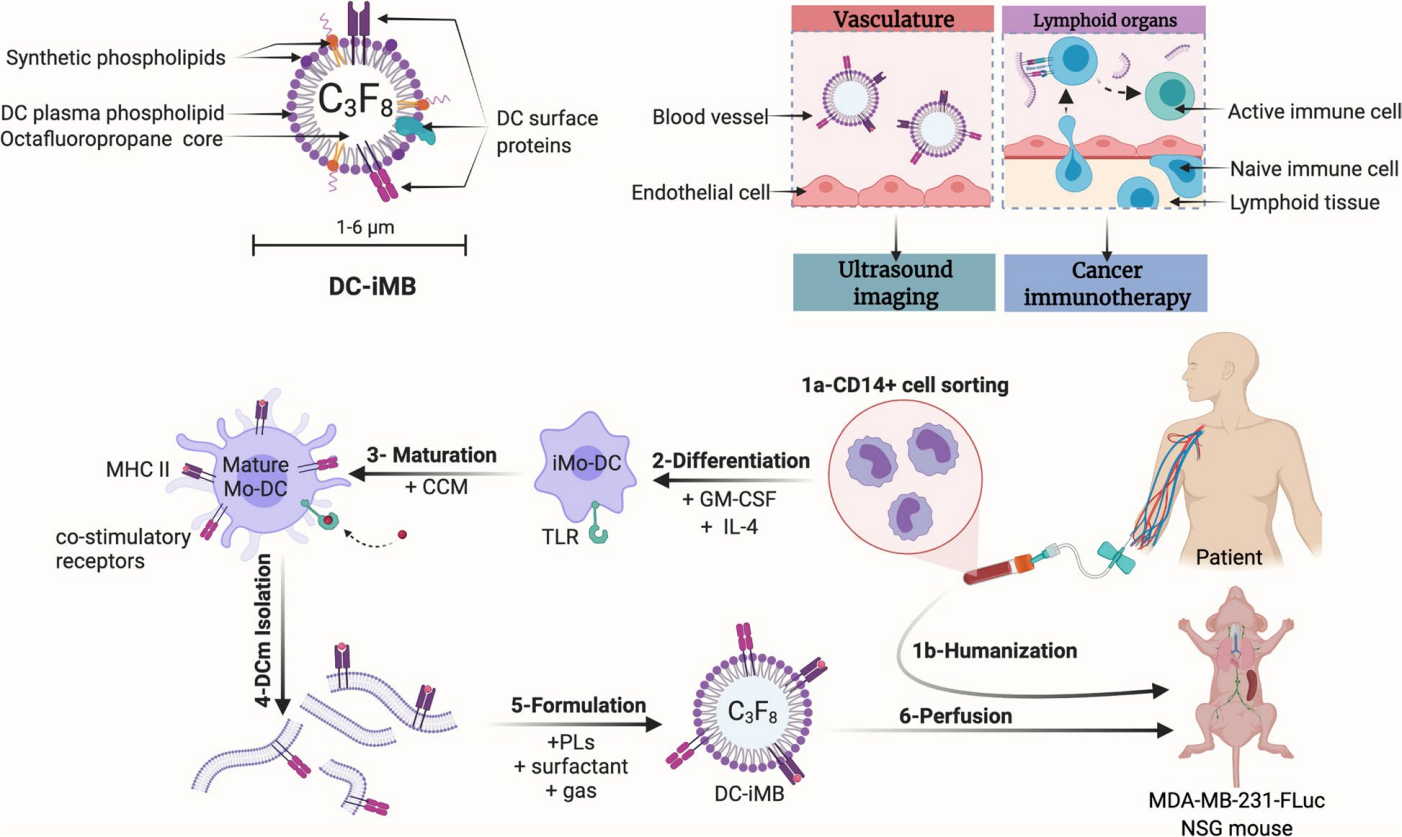
Personalized DC-iMB strategy for TNBC immunotherapy. **a** Schematic illustration of DC-iMB showing the presence of synthetic phospholipids, DC membrane phospholipids and proteins, and gas core. **b** By nature, DC-iMB allows US imaging (e.g.*,* tumor size and perfusion monitoring, spleen retention visualization), and targeted immunotherapy via TNBC specific antigen presentation and naïve T-cell activation in lymphoid organs (thymus, spleen, and lymph nodes). **c** DC-iMB vaccine preparation: Monocytes (CD14^+^) were isolated from patient’s peripheral blood for autologous DC generation (1a) while hPBMCs were used for human immune system reconstitution (i.e.*,* T-cell engraftment) of immunodeficient NSG mice (1b). Immature monocyte-derived DCs (iMoDCs) were generated by culturing the isolated CD14^+^ cells with GM-CSF and IL-4 (2). Monocyte-derived DCs (MoDCs) were matured and pulsed with MDA-MB-231 cancer cell derived membrane antigens (3). The plasma membrane from MoDCs was then isolated (4) and used for DC-iMB formulation (5) before injection into a humanized mouse model of TNBC via several cycles of intravenous (i.v.) injections (6). CCM: cancer cell membrane; DCm: DC membrane; GM-CSF: granulocyte macrophage colony stimulating factor; LNs: lymph nodes; MHC: major histocompatibility complex; PLs: phospholipids; TLR: Toll-like receptor.

TNBC is a breast cancer (BC) subtype with the most aggressive clinical course [26]. Compared to other BC subtypes, TNBC does not respond to currently available therapies targeting the estrogen receptor (ER) or the human epidermal growth factor 2-receptor (HER2). We hypothesized that DC-iMBs could offer a new way to deliver cancer vaccine by enhancing lymphoid organ homing and resident T-cell activation while allowing US molecular imaging to monitor their deliveries, and tumor response to treatment. In this study, we present the first DC membrane-based immunotheranostic MBs prepared ex vivo for TNBC therapy and show its therapeutic potential in a mouse model of TNBC **(**Scheme 1**)**. Significant differences between human and mouse immune systems and species-specificity preclude direct translatability [27]. To address this issue, one approach aims to re-create the human immune system in mice having little or no background immune system. In this instance, we used severe combined immunodeficient (SCID) mice, deficient in B and T lymphocytes and natural killer (NK) cells (NOD-scid-IL2rg^−/−^ (NSG) mice). This animal model is frequently used for various humanization processes including human peripheral blood mononuclear cells (hPBMCs), and CD34+ stem cell injection. We and others generated humanized immune system in SCID mice by engrafting functional human immune cells allowing for immune response investigation on various therapeutics including DC-based vaccines [28, 29]. We demonstrated DC-iMB efficacy in inducing anti-tumor immune response leading to an overall reduced tumor growth and tumor size, longer-term survival, and increased apoptotic events in tumor tissue of all treated animals. Moreover, we showed the possibility for real-time visualization of DC-iMB trafficking together with lymphoid tissue homing potentiating T-cells activation.

## Methods

### Tumor cell lines

The Human breast cancer cell line MDA-MB-231(ATCC HTB-26) was cultured in Dulbecco’s Modified Eagle Medium (DMEM) supplemented with 10% fetal bovine serum (FBS), 100 U/mL penicillin and 0.1% streptomycin (all from ThermoFisher Scientific, USA), and maintained in a 37 °C incubator with 5% CO_2_ and 95% air. Tumor cells were grown adherent and utilized in experiments when their cultures were 70% confluent. The cell line was regularly tested for any possible mycoplasma contamination (MycoAlert kit, Lonza, Allendale, NJ).

### Generation of human immature DCs

The hPBMCs were collected from buffy coats from healthy adult donors (Stanford Blood Center). Blood from 5 different healthy donors (blood type O+) were employed in the different experiments. The PBMCs were washed three times with PBS and counted using the trypan blue dye exclusion method with a hemocytometer. Human blood-derived monocytes were then sorted using anti-human CD14 MicroBeads (Miltenyi Biotec, Bergisch Gladbach, Germany), as per manufacturer’s protocol. Cell recovery was calculated as: % Recovery = (Total number of cells in CD14 enriched fraction) x (% Purity of enriched fraction)) / ((Total number of cells that will be separated) x (% Purity of staring fraction) × 100. The positively sorted CD14 cells were re-suspended in RPMI-1640 medium (ATCC 30–2001) supplemented with 10% FBS and 100 U/mL penicillin and 0.1% streptomycin and seeded at a density of 2 × 10^6^ cells per well in 6-well low adherence plates (Corning, Tewksbury; USA). Monocytes were cultured in a 37 °C, 5% CO_2_ incubator for 6 days with IL-4 (100 ng/ml), and GM-CSF (25 ng/ml), changing the medium every 2 days, to generate autologous iMoDCs.

### Priming of human DCs with TNBC cell line antigens

CCMs replicate the surface antigenic diversity of tumor cells, including the full array of known and unknown tumor-specific antigens (TSAs) and tumor associated antigens (TAAs). Therefore, CCMs can present a large antigenic spectrum to DCs, ultimately maximizing the likelihood of target identification by CTLs. In the aim of priming immature DCs ex vivo, we used the plasma membrane of MDA-MB-231 cells. MDA-MB-231 cells grown to 80% confluency were washed with 10 mL of PBS three times. For cell disruption, 10 mL of ice-cold distilled water was added and kept for 1 min. Cell disruption with ice-cold water application was repeated three more times. The plates were then washed twice with 10 mL of PBS to remove intracellular debris and the intact nucleus leaving the adherent cell membranes on the plate. After washing, the cell membrane was scraped and collected in 10 mL of PBS, briefly vortexed and centrifuged at 800×g for 5 min to remove any leftover intact nucleus. The supernatant was further centrifuged at 10,000×g for 20 min to remove any intact nucleosome, after which the supernatant was collected and centrifuged at 100,000×g for 60 min. The pellet containing the plasma membrane material was then washed once in 2 mL of PBS, and tested by western blot for cellular markers such as GAPDH, Histone 3, Cytochrome C, and N-cadherin. CCM protein concentrations were quantified using a bicinchoninic acid (BCA) assay kit (Thermo Fisher Scientific) and used for antigen pulsing of DCs. Then, cultured iMoDCs were stimulated for 24 h with CCMs (25 mg/mL). MoDCs, or simply “DCs”, were evaluated by flow cytometry using a panel of immune markers to assess correct differentiation and maturation processes. In the aim of using DC membranes to incorporate in DC-iMB formulation with enhanced lymphoid organ targeting and antigen presentation efficacy, we isolated the plasma membranes of mature DCs. Mature MoDC suspensions were collected and cryopreserved while adherent cells were washed with 10 mL PBS three times and used for DC membrane isolation using the same protocol used for CCM isolation from MDA MB231 cells. The DC plasma membrane material was stored at − 20 °C until use for DC-iMB preparation.

### Mice

NOD-scid-IL2rg−/− (NSG) mice were used for the study. The Administrative Panel on Laboratory Animal Care of Stanford University approved all procedures using laboratory animals used in this study and all experiments were conducted in accordance with the Guidelines for the Care and Use of Laboratory Animals.

### Murine subcutaneous breast tumor models

MDA-MB-231 cells were grown to 70% confluency, subsequently trypsinized, counted in a hemocytometer, and resuspended in 50 μL PBS mixed with 50 μL of Matrigel (BD Biosciences, San Jose, CA). MDA-MB-231 cells were inoculated subcutaneously (3 × 10^6^ cell/site) on the lower left and right flanks of female NSG mice (6 weeks old, Charles River, Wilmington, MA) 5 days before humanization protocol start. Mice were daily monitored, and tumor growth was assessed by digital caliper measurements in two dimensions and tumor volume was calculated as: Volume = 0.5 × (width)^2^ × (length). Animals were sacrificed when cumulated tumor volume was > 2000 mm^3^.

### Humanized immune system mice and vaccination schedule

Five days after tumor implantation, mice were randomized into 4 treatment groups: 1) hPBMCs + MBs; 2) hPBMCs + DC-iMBs; 3) hPBMCs; 4) untreated control (Supplementary Table S1). Two doses of hPBMCs were given to groups 1, 2 and 3, first at day 5 and second at day 7 post-tumor implantation. To keep an identical human leukocyte antigen (HLA) system and to avoid any mismatch, we used hPBMCs isolated from a single donor blood sample for mouse humanization (i.e.*,* doses 1&2) and for the generation of DCs (later used as DC-iMBs). Freshly isolated human PBMCs were used for the first dose. Cryopreserved hPBMCs were used for the second dose. In both cases, cells were washed three times in PBS by centrifugation at 350 xg for 5 min and resuspended in RPMI-1640 medium. Viable cells were enumerated using a hemocytometer. hPBMCs concentration was adjusted to 5 × 10^7^ cells/mL in RPMI-1640 medium. 0.2 mL (1 × 10^7^ hPBMCs) were injected intravenously into the lateral tail vein of each mouse in groups 1 to 3. Mice in group 4 were injected with 0.2 mL PBS using the same procedure. Animal’s body weight was recorded for signs of graft-versus-host disease (xGVHD) throughout the duration of the study. A small aliquot of peripheral blood from each animal (< 100 μL) was collected via the facial vein into K_2_EDTA tubes weekly for assessment of humanization by flow cytometry phenotyping. Blood samples from animals in the same group were pooled. On days 16, 19, 22, 26, 29 and 33, extemporaneously formulated particles (MB or DC-iMBs) were administrated in corresponding treatment groups. A total of 10^7^ particles were injected intravenously (100 μL) and the same volume of PBS was injected in animals from untreated groups (i.e.*,* groups 3 and 4).

### Flow cytometry

Cultured monocytes, iMoDCs, and MoDCs were stained with hCD11c (FITC), hCD14 (FITC), hCD33 (APC), hCD45 (PE), hCD80 (APC), hCD83 (FITC), hCD86 (PE), hCD206 (PE), hMHC II (FITC). Assessment of humanization was realized by staining mouse peripheral blood using hCD45 (PE), and mCD45 (PacBlue) after red blood cell lysis and PBMC recovery. Spleen, lungs, thymus, lymph nodes and tumors were excised and homogenized by mechanical dissociation and single cell suspensions were prepared by filtering through a 40-μm nylon cell strainer. Cell suspensions and blood samples were stained with hCD3 (PE-Cy7), hCD4 (APC), hCD8 (PacBlue), hCD83 (FITC), hMHC II (FITC). Antibodies used for flow cytometry were purchased from Biolegend (San Diego, CA). Data were obtained by flow cytometry (Guava easyCyte; Luminex Corp., Austin, TX) and analyzed using FlowJo software (Tree Star, Ashland, OR, USA).

### Microscopy

Changes in cell morphology during monocyte differentiation were visualized using bright field images on a Celigo S Imaging Cytometer (Nexcelom Bioscience, Lawrence, MA).

### Optical imaging

At predetermined time-points of the study, mice were anesthetized via a nose cone using 2% isoflurane in oxygen and intraperitoneally injected with D-luciferin (3 mg). Tumor bioluminescence was assessed using a Lago spectral instruments imaging system (Tucson, AZ).

### Spleen US imaging

NSG mice bearing MDA-MB-231 tumors were used for US molecular imaging using two MB constructs (MB and DC-iMB) and PBS for negative comparison. After mice were anesthetized with 2% isoflurane in oxygen, each mouse received a total of 10^7^ MBs or DC-iMBs (100 μL) by IV bolus injection via tail vein. The same volume of PBS was injected in control animals. All in vivo imaging studies were performed in contrast mode using a high-resolution US imaging system (Vevo 2100, FUJIFILM VisualSonics, Inc., Toronto, ON, Canada) with the transducer (MS250, VisualSonics; lateral and axial resolution of 165 μm and 75 μm, respectively) placed over the tumor area, guided by B-mode imaging to detect the target tissue of interest. Contrast mode images were acquired using an 18 MHz linear transducer (MS250), and all imaging parameters (focal length, 10 mm; transmit power, 4%; mechanical index, 0.2; dynamic range, 40 dB) were kept constant during and between all imaging sessions. Each experiment was performed for 5 minutes after MBs or PBS injection.

### MB preparation

For this study, we prepared two types of lipid-shelled bubbles: (1) control MBs and (2) DC-iMBs. DC-iMB preparation includes both DC plasma membrane and lipid solution processing. First, a lipid solution was prepared by dissolving a mixture of lipids comprising of DPPA (1,2-dipalmitoyl-sn-glycero-3-phosphate), DPPC (1,2-dipalmitoyl-sn-glycero-3-phosphocholine), and DSPE-MPEG-5000 (1,2-distearoyl-sn-glycero-3-phosphoethanolamine-N-methoxypolyethylene glycol)-5000] (Avanti Polar Lipids, Inc., Alabaster, AL) into sterile physiological saline solution at a molar ratio of 7:55:5 (0.75 mg total of lipid constituents). The lipid solution was further homogenized using a high shear fluid processor (LV1-microfluidizer, Microfluidics, Westwood, MA). We set the microfluidic system at 30,000 psi, washed the working-track five times with 75% ethanol solution, then re-washed three times with saline solution. We then injected the lipid solution into the system and extracted the solubilized lipid solution at the outlet. The microfluidic processing was repeated three times to homogeneously solubilize all the lipids. DC plasma membranes were isolated as mentioned previously, and the same procedure was applied for their solubilization in saline. Then, to form stabilized DC-iMBs, the processed lipid solution and DC membrane solution were mixed at a mass ratio of 75:25 in a 3 mL- headspace glass vial (Wheaton, Millville, NJ). The solution was supplemented with a non-ionic copolymer surfactant, Pluronic F-127 (0.03 mg/mL; Sigma-Aldrich, St. Louis, MO), glycerol (125 mg/mL; Sigma-Aldrich, St. Louis, MO) and propylene glycol (105 mg/mL; BioWorld, Dublin, OH). The vial was capped, sealed with a vial crimper, and stored at 4 °C. Upon usage, air was manually replaced by injecting octafluoropropane (C3F8, Fluoromed, L.P., Round Rock, TX) gas. Finally, the solution was activated by mechanical shaking using an amalgamator (VialMix shaker, Lantheus Medical Imaging, Inc., North Billerica, MA) for 45 s to generate the DC-iMBs. As a negative control, MBs were prepared using the same techniques detailed above but without adding DC plasma membrane.

### Dynamic light scattering (DLS), particle optical sizing device and scanning electron microscopy (SEM)

The mean hydrodynamic diameters and of MBs and DC-iMBs were measured using a single particle optical sizing device (0.5 to 400 μm measurable range, Accusizer 770A, Particle Sizing Systems, Santa Barbara, CA, USA). The zeta-potentials (surface charge) were measured at 25 °C by dynamic light scattering (DLS) with a scattering angle of 90° (Zetasizer Nano ZS90 sizing device, Malvern Panalytical Ltd., Malvern, U.K.) with samples dispersed in distilled water. Scanning electron microscopic (SEM, Zeiss Sigma) images were collected at 3000× magnifications with an accelerating voltage of 2.0 kV.

### Hematoxylin and eosin (H&E) staining

At the termination of the study, mice were sacrificed, major organs and tumors harvested and fixed in 4% paraformaldehyde at 4 °C for 24 h. Organs and tumors were then immersed in 70% ethanol, embedded in paraffin, and sliced at 5 μm thickness using a Leica cryo-microtome (RM2255, Leica, GE). These sections were stained in hematoxylin (Sigma-Aldrich) for 2 min, rinsed with water, and transformed in 1% HCl acid/alcohol for 30 s. The slices were washed and immersed in bluing solution (Thermo Fisher Scientific) for 1 min, washed with water and rinsed in 10 dips of 95% ethanol. The slides were then counterstained in eosin by dipping into ethanol diluted eosin (Thermo Fisher Scientific) solution (ethanol:eosin = 1:5) for 20 seconds, dehydrated using 95% alcohol, then xylene for 5 min, each. Finally, the slides were mounted with xylene-based mounting medium (Permount, Sigma-Aldrich) and imaged using a Nanozoomer system (Hamamatsu, Japan).

### Ex vivo immunofluorescence staining

We analyzed the presence of CTLs and helper-T-cells in the spleen by confocal microscopy. Collected spleens were fixed and cryosectioned into 10 μm slices. Spleen sections were incubated in a blocking solution of 2% bovine serum albumin and 1% normal donkey serum (both from Sigma, St. Louis, MO, USA) for 60 min at room temperature in a humidifying chamber. The slices were stained for CD4 and CD8 markers (BioLegend, San Diego, CA) diluted in incubation buffer at 4 °C overnight. All tissue slides were then washed in PBS and supplemented with 100 μL of a Hoechst solution for 5 min at room temperature. Excess dye was removed by PBS and water washes. Slides were mounted using an anti-fade mounting media (Vector Laboratories, Burlingame, CA) for confocal fluorescence microscopy visualization. We imaged tissue slices using a Leica TCS SP8 laser confocal microscope.

### Apoptosis TUNEL assay

To analyze the therapeutic effect of DC-iMB delivery, apoptotic cell frequency was analyzed. TUNEL staining was done on tumor sections with an in situ apoptosis detection kit (TACS; Trevigen, Gaithersburg, MD), according to the manufacturer’s instructions. In brief, immobilized tissue samples were washed with PBS and incubated with 50 μl Cytonin solution for 1 h. The slides were then washed two times in deionized water, 2 min each, followed by washing in immerse sample in Quenching Solution for 5 min. The samples were then washed in PBS for 1 min and immersed in 1X Terminal deoxynucleotidyl transferase (TdT) Labeling Buffer for 5 min. The samples were then covered with 50 μl of Labeling Reaction Mix and incubated for 1 h at 37 °C in a humidity chamber. The samples were then immersed in 1X TdT Stop Buffer for 5 min, and washed two times in deionized water, 5 min each, before being covered with 50 μl of Strep-HRP solution and incubated for 10 min at 37 °C. Then, samples were washed two times in 1X PBS, 2 min each, immersed in DAB Solution for 5 min, and washed two times in deionized water, 2 min each. Finally, samples were immersed in 1% Methyl Green for 30 s and gradually dehydrated by dipping slides ten times each in 2 changes of deionized water, 70 95, and 100% ethanol. Tissue samples were covered with a glass cover slip and mounting media and imaged using Nanozoomer digital slide scanner (Hamamatsu, Japan). TUNEL-positive cells in four different fields per sample were counted, and results were expressed as % of apoptosis area per mm^2^ of tissue section.

### Statistical analysis

All statistical analysis was performed using Student’s t test and Prism software (Version 8.4.1, GraphPad, LLC). Results were presented as mean ± standard deviation (SD). The results were considered statistically significant when the corresponding *p*-value was < 0.05.

## Results

### hPBMC isolation and CD14+ cell enrichment by magnetic-activated cell sorting (MACS)

**Fig. 1:**
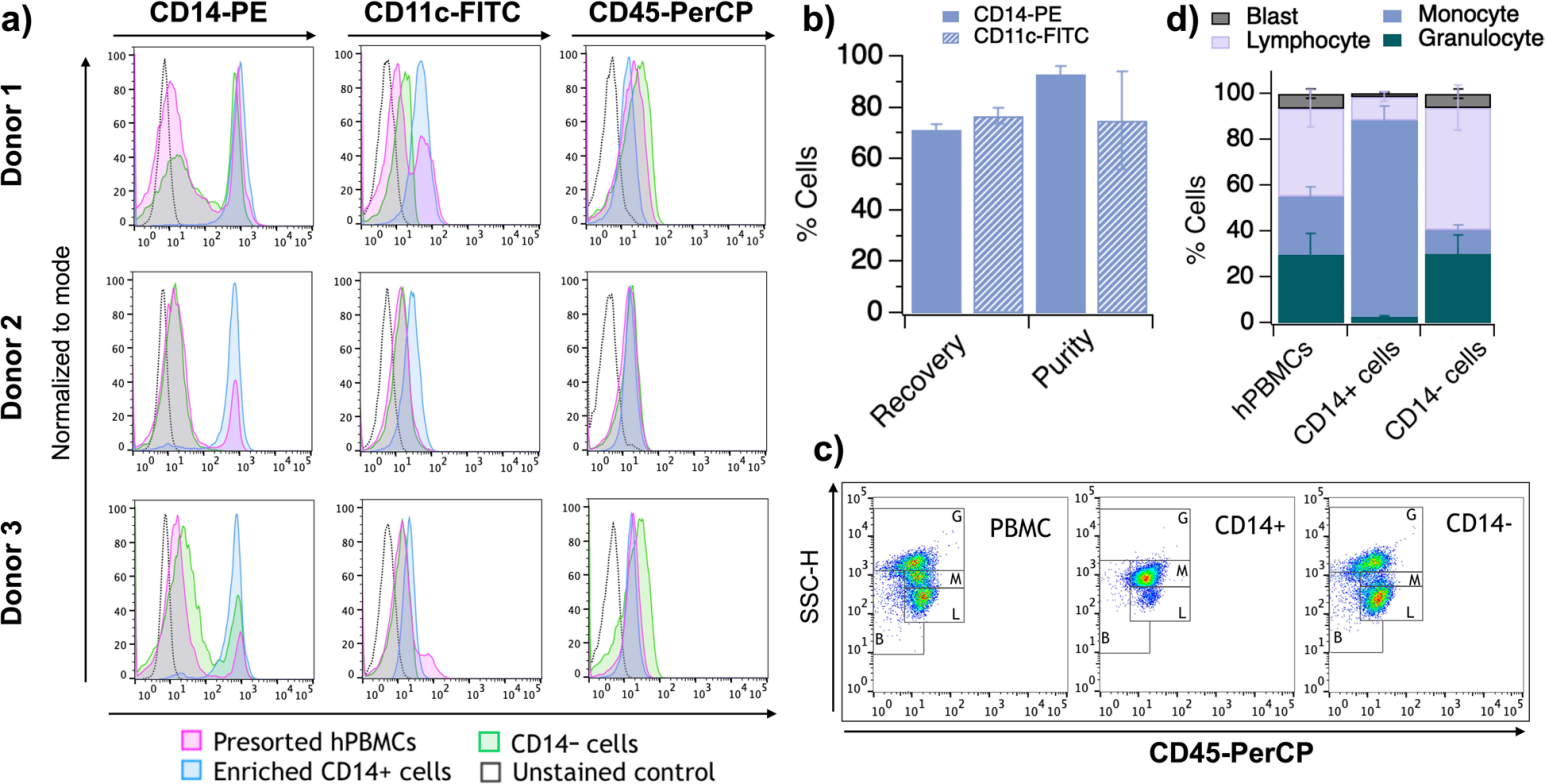
CD14-positive cell enrichment from 3 healthy blood donors and their characterization for different surface markers. **a** Histogram plots showing CD14, CD11c, and CD45 marker specific fluorescence signals from cells pre- and post- cell sorting. **b** CD14^+^ sorted cell fraction purity and recovery quantification. **c** hPBMCs gating strategy in CD45 vs. SSC-H dot plot. **d** Percentage assessment of each major cell group. All data are shown as Mean ± SD. G = granulocyte; M = monocyte; L = lymphocyte; B = blast

The monocytes of healthy blood donors were sorted in parallel from hPBMCs using CD14^+^ conjugated magnetic microbeads by following Miltenyi magnetic separation protocol. Cell counting indicated that 7 × 10^7^ ± 19 × 10^6^ CD14^+^ cells could be isolated from 7 × 10^8^ ± 30 × 10^7^ PBMCs (approximately 10%; consistent with percentages reported in the literature for healthy individuals) [30]. The cell surface expression markers CD14, CD11c, and CD45 were assessed pre- and post- CD14-positive cell sorting (Fig. 1a, Supplementary Fig. S1). CD14 was detected with 93% ± 3 purity in the sorted cell population with recovery of 65% ± 6. Good reproducibility between all donors was observed (Fig. 1b), and sorted cells primarily consisted of monocytes (Fig. 1c, d). Sorted CD14-positive cells also increased the enrichment in CD11c-positive population (75% ± 19 purity and 75% ± 7 recovery), consistent with monocyte molecular profile. Moreover, analysis of the leukocyte common antigen CD45-positive population showed good purity of hPBMC (79% ± 5) comprising 38% ± 8 of lymphocytes, adequate for mouse humanization. Finally, a similar CD45-positive population was observed in the CD14-enriched fraction (88% ± 6; *p* = 0.117) (Supplementary Fig. S1).

### Human monocytes differentiate and maturate ex vivo into TNBC-pulsed MoDCs

**Fig. 2:**
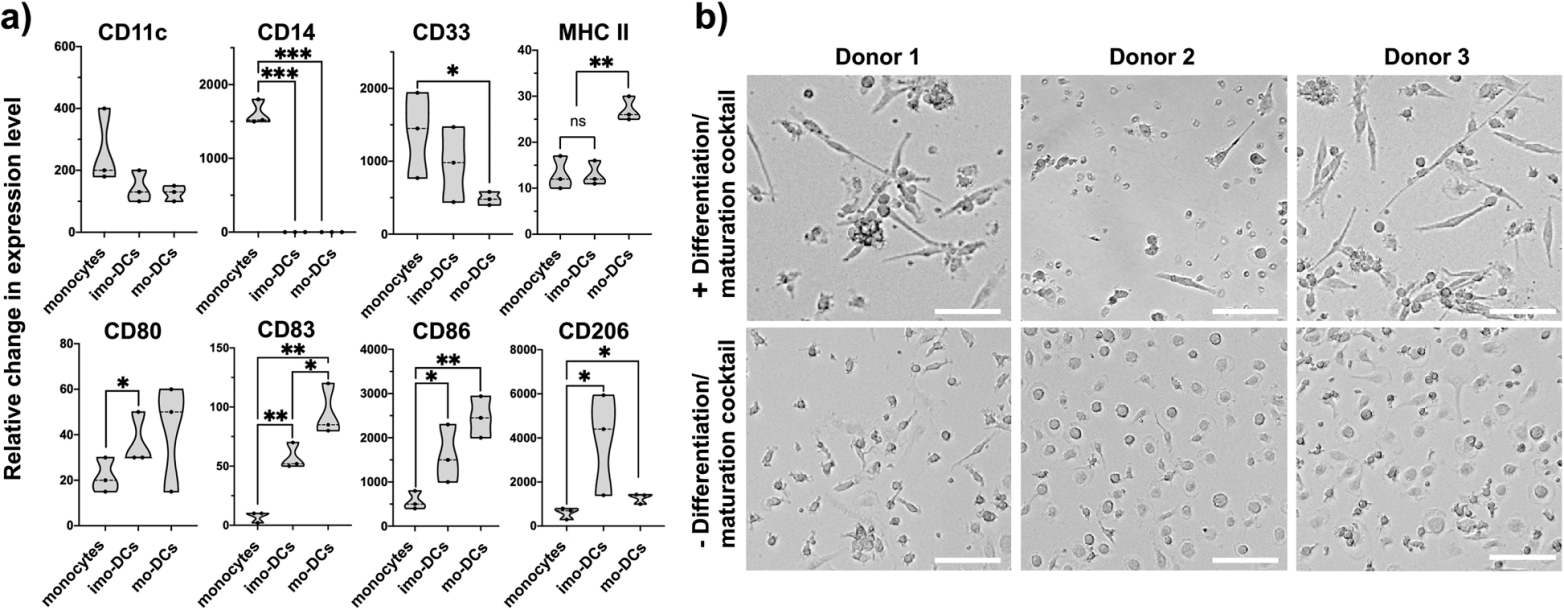
MoDC maturation and activation ex vivo. **a** FACS analysis of various cell surface markers (CD11c, CD14, CD33, MHC II, CD80, CD83, CD86 and CD206). Fluorescence intensity shift was compared to unstained control. **b** Cell morphology after complete maturation and activation (+) compared to control (−). Cells were photographed using a digital camera assembled on a bright field inverted microscope. Original magnification was 40×. Scale bar = 100 μm.*p < 0.05; **p < 0.005; ***p < 0.0001

A key step in DC-based vaccine preparation is their maturation and pulsing with cancer antigens. First, MDA-MB-231 cancer cells were cultured separately, and the plasma membranes were isolated by differential centrifugations. We confirmed the CCM fraction purity by western blot analysis using a series of intracellular and plasma membrane protein markers (Supplementary Fig. S2). The plasma membrane-specific marker, N-Cadherin, was detectable in the CCM purified fraction. Primarily located in the cytosol, GAPDH can also insert into plasma membranes as an anchor protein. Accordingly, we observed some GAPDH signal in the CCM fraction. Conversely, there was no evidence of intracellular marker Cytochrome C in the CCM fraction, nor nuclear Histone H3, indicating no mitochondrial nor nuclear contamination, respectively. To monitor the ex vivo maturation process of DCs and ensuring high immunogenicity for later use in vivo, we determined the surface immunophenotype of DCs cultured with CCMs isolated from MDA-MB-231. Variation of surface markers between DC differentiation/maturation stages, including expression levels of CD11c, CD14, CD33, CD45, MHC II, CD80, CD83, CD86, and CD206, were then analyzed by flow cytometry and fluorescence intensity shifts were quantified **(**Fig. 2a, Supplementary Fig. S3). Monocytes cultured with only differentiation factors presented a significant down-regulation in CD14 expression level (1600-fold; *p* < 0.0001), expressed significantly more CD80, CD83, and CD86, (1.7-fold; *p* < 0.05, 7.8-fold; *p* < 0.005, and 2.8-fold; *p* < 0.05, respectively), and a significantly increased level of CD206 (6.5-fold; *p* < 0.05) compared to untreated monocytes. These immunophenotyping results confirmed the differentiation of monocytes into iMoDCs upon GM-CSF and IL-4 stimulation. Furthermore, cells cultured with full differentiation and maturation cocktail demonstrated a higher expression of the co-stimulatory protein CD86 (93% ± 8.8) compared to undifferentiated cells (69% ± 21) and pre-matured cells (77% ± 13). Stimulated cells also presented a significantly up-regulated level of the DC activation marker CD83 compared to untreated monocytes (14.7-fold; *p* < 0.005) and iMoDCs (2.1-fold; *p* < 0.05), and of MHC-II (2.2-fold; *p* < 0.005) compared to both untreated monocytes and iMoDCs. Besides, mature MoDCs lost the expression of CD14 and presented a significant down-regulation of CD33 (2.8-fold; *p* < 0.05). Expression level of the mannose receptor for antigen uptake, CD206, decreased during DC maturation likely because of receptors internalization during the maturation process. CD11c level was not significantly affected by differentiation/maturation stages. Overall, our ex vivo assessments confirmed that MoDCs displayed suitable phenotype for the induction of anti-tumor immunity. Moreover, cells stimulated with full differentiation/maturation cocktail showed long cytoplasmic veils (e.g.*,* dendrites) typical of mature DCs (Fig. 2b). Conversely, non-induced monocytes presented a rounded shape, confirming the immature state.

### In vitro characterization of DC-iMBs

**Fig. 3:**
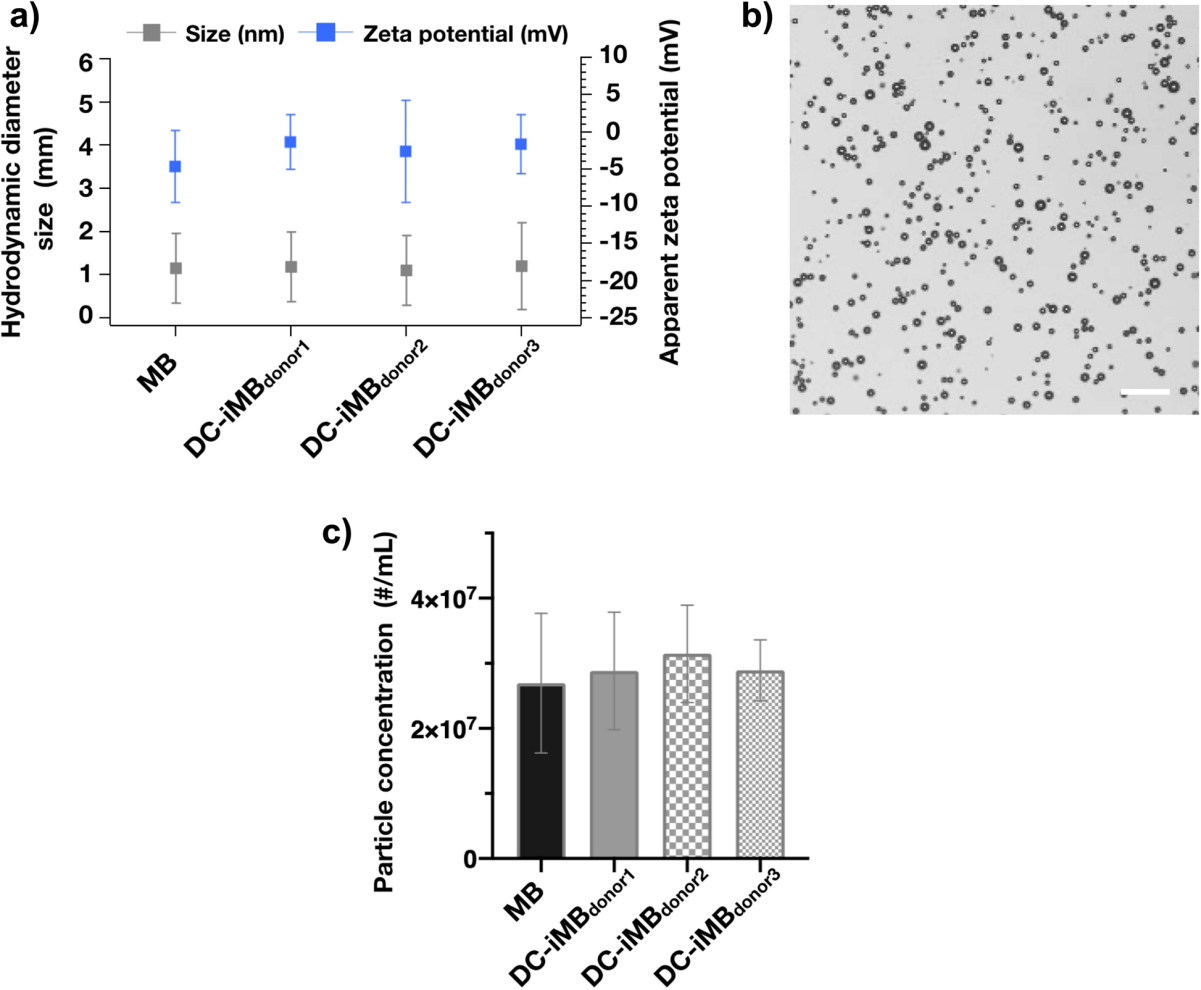
In vitro DC-iMB characterization. **a** Diameter size distribution and zeta potential of MB and DC-iMB_donor 1,_ DC-iMB_donor 2,_ and DC-iMB_donor 3_; **b** Bright-field microscopic image of DC-iMBs. Scale bar is 50 μm; and **c** Particle concentration after formulation

After pulsing the MoDCs using CCM antigens, we isolated their plasma membranes to prepare DC-iMB using microfluidic-based reconstitution strategy. The incorporation of MoDC plasma membranes as part of DC-iMB composition did not affect the bubble mean diameter (1.18 ± 0.8 μm, 1.10 ± 0.8 μm, 1.21 ± 1.0 μm, from donors 1, 2 and 3, respectively) compared to control MB (mean diameter = 1.15 ± 0.8 μm) **(**Fig. 3a, Supplementary Fig. S4). The zeta potentials were − 1.5 ± 3.6 mV, − 2.7 ± 6.8 mV, − 1.8 ± 3.9 mV, for donors 1, 2 and 3, respectively, compared to − 4.7 ± 4.8 mV for control MB. The negative charges of commercial PLs constituting both MBs and DC-iMBs prevent bubbles from aggregation, while the hydrophilic character keeps them water dispersible. A bright-field microscope image of DC-iMBs is shown in Fig. 3b. MB and DC-iMB concentrations were similar upon preparation with good reproducibility between donors (Fig. 3c). Overall, our in vitro results are consistent with the characteristics of clinically used MBs [31]. In addition, we examined the bubble’s surface morphological changes during cell membrane impregnation using scanning electron microscopy (SEM) (Supplementary Fig. S5). While control MB showed a smooth surface, plasma membrane impregnation induces pronounced morphological changes of the shell. The surface appeared rough with randomly distributed membrane fragments onto the shell.

### Human immune system reconstitution in mice through hPBMC injections

To develop a humanized immune system model to study DC-iMB vaccination in vivo*,* we used NSG mice xenografted with hPBMCs. Female NSG mice were randomly divided into 4 groups namely: 1) hPBMCs + MBs; 2) hPBMCs + DC-iMBs; 3) hPBMCs; and 4) untreated control, (*n* = 4/group). The human immune system was reconstituted in animals from groups 1 through 3 by intravenous injection of 10^7^ hPBMCs twice over 7 days while animals from control group 4 received PBS. Twelve days after hPBMC or PBS injection, a small volume (< 100 μL) of peripheral blood from each animal belonging to the same group was collected and pooled. Freshly isolated hPBMCs from a healthy blood donor were used as control. PBMCs were gated and analyzed for human and mouse CD45 expression levels to assess immune cell engraftment. FACS results demonstrated a rapid and successful hPBMC engraftment in NSG mice from groups 1, 2 and 3 with approximatively 16 ± 5.3% hCD45-positive PBMCs (Supplementary Fig. S6).

### In vivo efficacy of DC-iMB as cell-free anti-TNBC vaccine

**Fig. 4:**
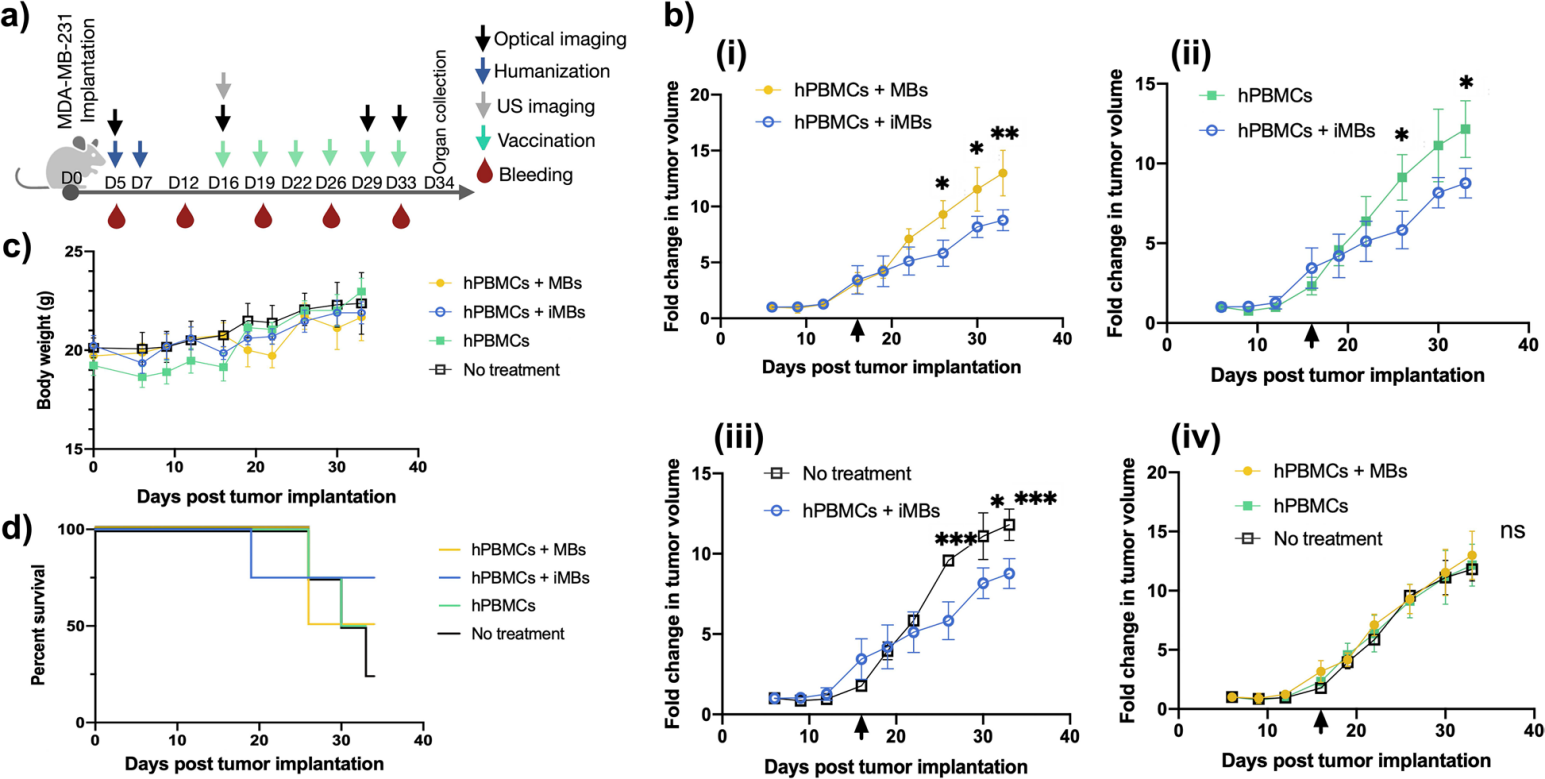
Therapeutic evaluation of DC-iMBs in TNBC bearing humanized immune system mice. **a** Schematic outline of the experimental design and timeline adopted for treatment, imaging, and blood collection. **b (i-iv)** Relative change in tumor volume over time. Black arrows indicate the starting date of therapeutic treatments. **c** Animals from different treatment groups measured for body weight over time (*n* = 4/group) to monitor the impact of treatments on animal health as well as xGVHD development. **d** Survival curves of animals from different treatment groups. All data are shown as Mean ± SD. *p < 0.05; **p < 0.005; ***p < 0.0001

**Fig. 5:**
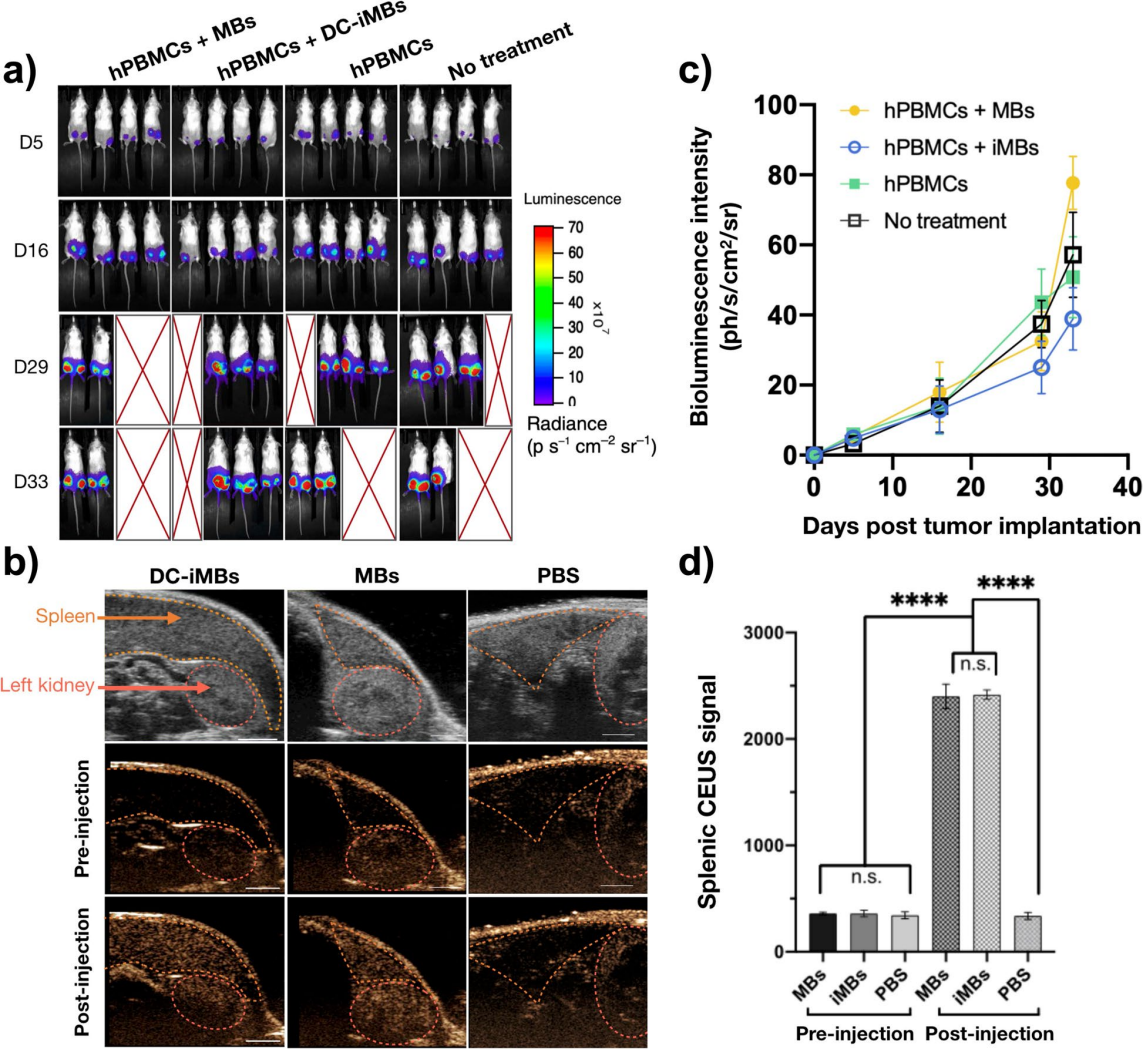
In vivo imaging evaluation of DC-iMB. **a** Bioluminescence images acquired at multiple time points during the treatments to assess therapeutic responses. **b** Representative B-mode US (top) and non-linear contrast images of a spleen pre-injection (middle) and post-injection (bottom) with DC-iMBs, conventional MBs, or PBS. Scale bar = 3 mm. **c** Quantification of tumor bioluminescence signal over time. **d** Quantification of US signal enhancement pre- and post- MBs, DC-iMBs, or PBS injection. CEUS: contrast enhanced ultrasound. ***p < 0.0001

Therapeutic efficacy of DC-iMB was evaluated in a xenograft mouse model of TNBC featuring the human immune system. We used NSG mice bearing MDA-MB-231 tumors on both lower flanks expressing firefly luciferase green fluorescence protein (FLuc-GFP) fusion reporter protein to monitor anti-cancer response by bioluminescence imaging. Five days post tumor implantation, mice were randomized into 4 treatment groups as described above. The treatment and imaging schedule used for the study is presented in Fig. 4a. Injection of DC-iMB, MB or PBS started on day 16. Tumor growth rate showed no significant difference in all 4 groups from day 0 through day 19 (Supplementary Fig. S7a, Supplementary Table S2), corresponding to the pre-treatment period (days 0 to 16) and early post-treatment (days 16 to 19) (Fig. 4b). However, starting day 19 until the end of the study, tumors of animals treated with DC-iMBs demonstrated a significant reduction in growth rate compared to those treated with control MBs or PBS (from 1.6 to 1.8-fold, *p* < 0.0001). Mouse body weights in all groups showed a gradual upward trend possibly owing to the increasing tumor weight as tumor growth progressed (Fig. 4c). Although no loss in body weight was observed, all PBMC treated animals presented first signs of xGVHD starting day 29 and forced us to terminate the study by day 34. Animals treated with DC-iMBs achieved the longest survival period, despite one death on day 19 likely unrelated to the treatment (Fig. 4d). Of note, we controlled that the immune system engraftment did not impact on tumor growth during the entire study length by comparing the hPBMC-treated group 3 and the untreated control group 4. Whole body optical images further indicated that DC-iMB-treated animals showed the lowest mean bioluminescence signal among all groups (Fig. 5a,c). Finally, DC-iMB was tested by molecular US imaging for its contrast enhancement capacity as well as its ability to circulate in the spleen and compared to conventional MBs (Fig. 5b,d). The spleen was first located on B-mode imaging. Then, MBs, DC-iMBs, or PBS were injected intravenously, and signal enhancements were followed using non-linear contrast mode. Upon injection of MBs and DC-iMBs, the US signal significantly increased in the spleen with similar enhancements (5.6-fold). PBS-injected mice were used as negative control.

### Ex vivo analysis of DC-iMB therapeutic effect

**Fig. 6:**
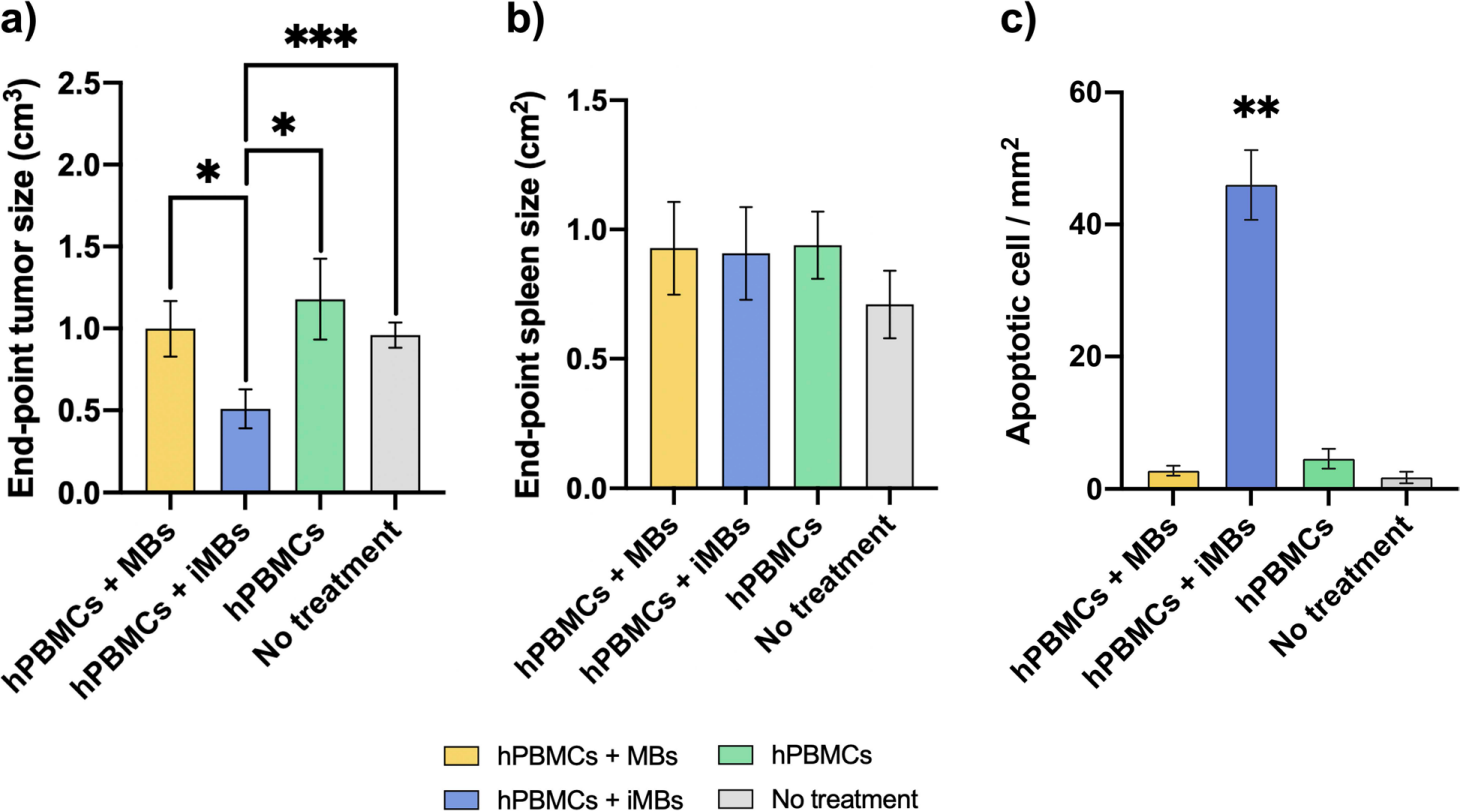
Terminal ex vivo size evaluation and apoptosis. **a** Tumor volume, and **b** spleen size measurements at study endpoint; **c** mean number of apoptotic cells per mm^2^ of tumor tissue. *p < 0.05; **p < 0.005; ***p < 0.0001

At study termination, mice were sacrificed, and major organs and tumors were harvested for histological analysis. Ex vivo tumor size measurements revealed to be significantly reduced in DC-iMB treated animals compared to all the other groups (1.9- to 2.3- fold) (Fig. 6a, Supplementary Fig. S7c). Mild splenomegaly was observed on spleens from humanized groups 1 to 3 compared to the untreated group 4 (1.3-fold), potentially due to the proliferation of human leukocytes within the white pulp, however results were not significantly different (Fig. 6b, Supplementary Fig. S7b). No sign of MB induced-toxicity was observed on H&E-stained sections from major organs (Supplementary Fig. S8a), however, tumor tissues treated with DC-iMB showed a significant increase in apoptotic events (Fig. 6c, Supplementary Fig. S8b).

**Fig. 7:**
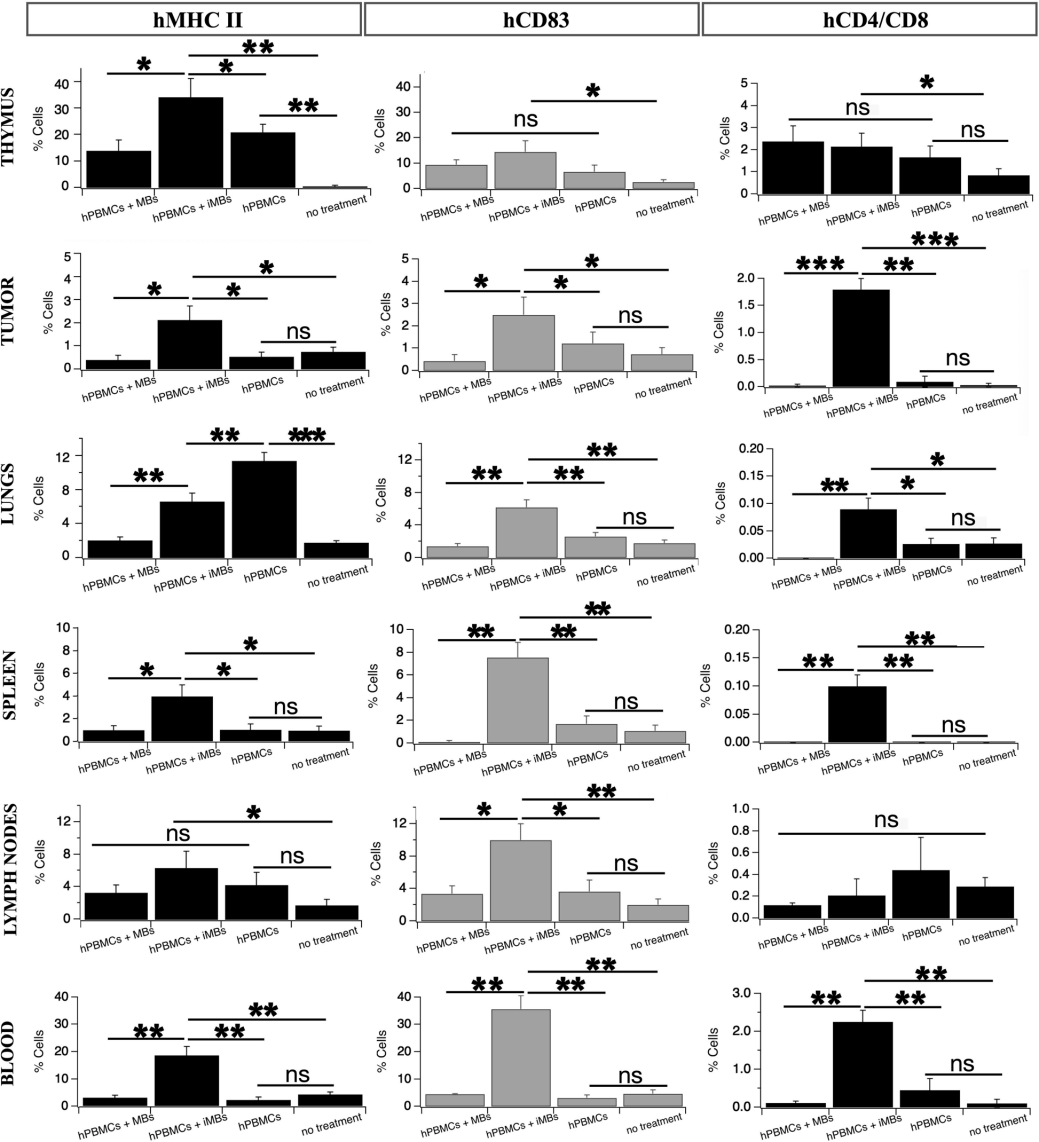
Ex vivo cell distribution in **a** spleen, **b** thymus, **c** tumor, **d** blood, **e** lymph nodes, and **f** lungs of mice treated or not with DC-iMBs. *p < 0.05; **p < 0.005; ***p < 0.0001

Primarily expressed on fully mature DCs, CD83 is also distributed among B and T lymphocytes after activation [32]. Our leucocyte FACS analysis revealed that all collected tissues from DC-iMB-treated mice demonstrated significantly increased levels of CD83^+^ cells (+ 6.6% in spleen (*p < 0.005*), + 12% in thymus (*p < 0.05*), + 1.8% in tumor (*p < 0.05*), + 4.4% in lungs (*p < 0.005*), + 31% in blood (*p < 0.005*), + 8% in lymph nodes (*p < 0.005*)) relative to the corresponding tissues from the untreated group (Fig. 7, Supplementary Fig. S9). Similar expression levels of hCD83 in tissues of humanized animals with or without conventional MB injections (*p > 0.05*). Importantly, a significant increase in hCD83 expression was found in the spleen (+ 6%, *p < 0.005*), tumor (+ 1.5%, *p < 0.05*), lungs (+ 4.2%, *p < 0.005*), blood (+ 31%, *p < 0.005*), and lymph nodes (+ 6.5%, *p < 0.05*) of DC-iMB treated animals compared to MB or PBS treated ones. Of note, the thymus is a primary lymphoid organ critical for the development of lymphocytes. Moreover, human T-cells can develop in humanized NSG mice in the thymus [33]. CD83^+^ cells in the thymus of all humanized animals were not significantly different (*p > 0.05*) and up 7.5% on average compared to the untreated group (*p < 0.05*), representing the basal pool of human engrafted active immune cells. Thus, our results indicated that vaccination by DC-iMBs triggered engrafted immune cell maturation and proliferation in the thymus before migrating via the blood circulation towards secondary lymphoid organs as well as in the tumor. Furthermore, all tissues from DC-iMB-treated animals presented significantly increased expression level of MHC II (+ 3.1% in spleen (*p < 0.05*), + 34% in thymus (*p < 0.005*), + 1.4% in tumor (*p < 0.05*), + 3.7% in lungs (*p < 0.005*), + 13% in blood (*p < 0.005*), + 4.5% in lymph nodes (*p < 0.05*)) compared to untreated animals. Importantly, we observed a significant increase in MHC II in the spleen (+ 3%, *p < 0.05*), tumor (+ 1.7%, *p < 0.05*), blood (+ 15%, *p < 0.005*), and thymus (+ 17%, *p < 0.05*) of DC-iMB treated animals compared to MB or PBS treated ones. Those results further indicated a positive response of APCs upon vaccine administration, with enhanced migratory and tumor infiltration properties. Once activated, T-cells (either CD4^+^ or CD8^+^) are crucial in achieving an anti-cancer immune response by effector and memory cells. Thus, we evaluated the expansion of T-cells in tissues of vaccinated mice. DC-iMB treated tumors revealed a surge in double positive CD4^+^CD8^+^ T -cells in the spleen (+ 0.1%, *p < 0.005*) whereas other groups failed in expressing them. We also found greatly increased levels of CD4^+^CD8^+^ T -cells in the tumor (+ 1.7%, *p < 0.005*), lungs (+ 0.07%, *p < 0.05*), and blood (+ 2%, *p < 0.005*) of DC-iMB treated animals compared to marginal levels of both T-cell types in the other groups. This strongly suggested the recruitment of TILs upon T-cell vaccine recognition. These data suggested that naive engrafted T-cells were activated upon interaction with DC-iMB in the spleen and were able to extravasate into the tumor microenvironment. T-cell reservoirs in the thymus and lymph nodes were not significantly different between all humanized groups.

**Fig. 8:**
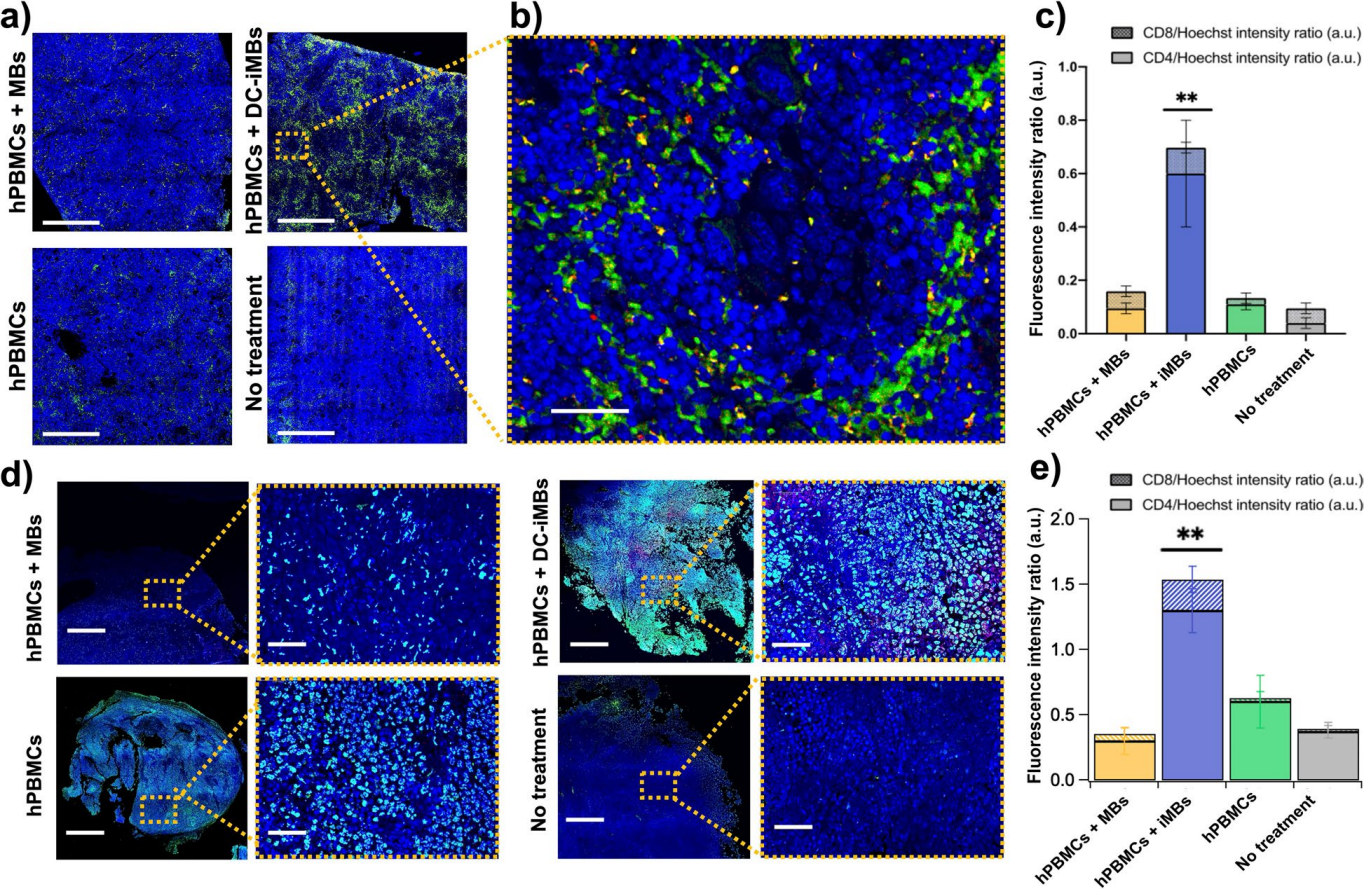
In situ characterization of CD4^+^ and CD8^+^ T-cell migration by confocal microscopy. **a** Representative spleen images. Tissues were processed for histologic analysis and triple stained with hoechst33342 (blue), hCD4 (green), and hCD8 (red). Scale bar = 0.5 mm; **b **Magnification of spleen area treated by hPBMCs + DC-iMBs. Scale bar = 40 μm; **c** Splenic quantification of fluorescence intensity ratios (hCD4/Hoechst and hCD8/hoechst33342) for all animal groups; **d** Representative tumor sections with respective magnified areas. Scale bars = 0.5 mm and 40 μm, respectively. **e** Quantification of fluorescence intensity ratios (hCD4/Hoechst and hCD8/hoechst33342) in tumor sections for all animal groups. **p < 0.005

Finally, to determine the presence and location of human lymphocytes, in situ immunofluorescence microscopy analysis of the spleen was performed (Fig. 8a-c). Although only vestigial germination centers were present, our results indicated high numbers of CD4^+^ T-cells in the spleen of DC-iMB-treated animals compared to all the other groups, which was consistent with our flow cytometry analysis. Further analysis of multiple tumor tissue sections indicated that tumors from DC-iMB-treated animals presented a significant increase in CD4^+^ (2.2–4.3-fold, *p < 0.005*) and CD8^+^ T cells (4.3–10.9-fold, *p < 0.005*) compared to the other groups (Fig. 8d-e).

## Discussion

The success of ICIs against selected solid tumors as well as ACTs has revolutionized cancer therapy landscape in the last decade [34]. However, in TNBC, only ~ 3.5% of patients [35] respond to ICIs with objective response rate (ORR) ~ 5–24% [36] due to immunoresistance mechanisms including extremely low tumor-infiltrating lymphocytes (TILs; applicable to 10–70% patients [37]), deregulation of immune checkpoints, and loss of tumor antigens [16]. Moreover, severe-to-fatal side effects remain a major concern for ICIs/ACTs due to uncontrolled autoimmune responses [38, 39]. This has amplified the need to assess alternative or combinatorial therapy options that can be beneficial for most patients not responding to ICIs/ACTs [37].

Anti-cancer DC vaccines aim to trigger a patient’s immune response against specific tumor antigens to regain control over tumor growth in a specific and long-lasting fashion. An example of DC vaccine that has been granted regulatory approval by the FDA is Sipuleucel-T for the treatment of metastatic castrate resistant prostate cancer [10]. Current DC vaccines have demonstrated high successes in pre-clinical models but have achieved modest ORR in patients (~ 8–15% based on RECIST (Response Evaluation Criteria in Solid Tumors) [40]. Nevertheless, the median overall survival has increased by at least 20% in many studies. Moreover, clinical evidence has shown that DC vaccine treated patients experienced antigen-specific CTL activity with increased NK cell [41].

Current anti-cancer vaccines are based on a single TSA or TAA, thus providing highly focused immunity in patients. Although encouraging in some melanoma [42] and prostate cancer [10], single-antigen vaccination often provides insufficient immunogenicity when facing the high heterogeneity and mutation rate of cancer cells [43, 44]. In addition, they likely promote immune escape and antigen loss [45]. Finally, target identification and selection remain a major clinical challenge as suitable targets can greatly vary between patients [46]. Importantly, due to its lack of known tumor targets, single antigen-based vaccines are currently absent for TNBC. One strategy to address these issues implies using whole cancer cells, whole cancer cell lysates (WCLs), or CCMs as multi-antigenic sources for DC priming. In contrast to bottom-up synthesis techniques, this top-down approach does not require prior identification of individual antigens. Thus, this is particularly adequate for TNBC. Whole cancer cell and WCL can be prepared in fewer steps and can prime the immune system against the complete antigenic profile of tumor cells [43]. However, the presence of a large amount of non-tumor-related antigenic material can cause significant decrease in immune response efficacy [47]. Importantly, CCMs replicate the entire surface antigenic diversity of the source tumor cells (all TSAs and TAAs). CCM-pulsed DCs can maximize the benefits of multi-antigenic vaccines regarding target identification with low susceptibility to immune escape. Moreover, enhanced immune response has been shown compared to whole cancer cell and WCL-pulsed DC vaccines [48–50]. Importantly, DC membrane vesicles were proven to carry 10- to 100-fold more TAA-MHC II complexes compared to whole DCs with the capability of initiating potent antitumor immunity [51–54]. Of note, DC membranes demonstrated a conserved spectrum of adhesion molecules and costimulatory molecules, including ICAM-3, CD40, CD44, and integrins promoting T cell interaction [55]. In addition, the DC membrane can also be guided towards lymph node through the lymph node homing CCR7 receptor molecule.

Here, we introduce the first cell-free DC vaccine in the form of immune-MB to mediate cancer immunotherapy. We tested its capacity in vitro and in a humanized immune system mouse model of TNBC. Immunophenotyping analysis of cultured human-derived monocytes confirmed correct differentiation into autologous MoDCs. Moreover, mature MoDCs presented enhanced expression of costimulatory molecules necessary for T-cell response. Importantly, immature MoDCs are known to fail to induce antigen-specific responses and may induce the differentiation of regulatory T-cells. Therefore, controlling that MoDCs have achieved full maturation and activation state prior to vaccine administration is critical to avoid inducing immune tolerance [56]. Interestingly, immature DCs have been used to promote anergic T-cell development in the settings of transplantation and automimmunity [57]. Similarly, DCs lacking the expression of costimulatory molecules have even engineered for immunosuppression purposes [58]. Ex vivo MoDC pulsing using homologous TNBC cell membranes provides the possibility of applying a wide spectrum of potential antigens that may not be reached in vivo and could bypass issues of tumor antigen loss. DC-iMBs were formulated from plasma membranes from mature MoDC incorporated into US contrast imaging microbubbles. Particle mean diameters, surface charges, and concentrations were similar to what was reported for conventional MBs used in the clinic. SEM images provided a direct visualization of DC-iMB surface morphology. In vivo*,* humanized animals who received DC-iMB treatment had the longest survival time and demonstrated a significantly slower tumor growth rate compared to the other groups treated with either control MBs or PBS. These results agreed with our bioluminescence imaging findings. Although no loss in body weight was observed during the study, PBMC treated animals presented signs of xGVHD starting day 29 post tumor cell injection and forced to end the study on day 34. Finally, DC-iMB tested for molecular US imaging demonstrated high signal enhancement in the spleen thereby offering real-time trafficking visualization and splenic passive accumulation as published elsewhere [22, 23]. Upon tumor resection, DC-iMB treated group presented a significantly smaller average tumor size while spleen sizes were similar between all groups. Analysis of apoptosis in tumor sections further confirmed the therapeutic effect of DC-iMB. Surface marker analysis indicated an increase in CD83+, MHC II+ and T-cells in most of the DC-iMB treated tissues. Control MBs did not appear to influence the immune system of humanized mice. Overall, DC-iMBs demonstrated capabilities for cross-presentation with T-cells and provided co-stimulatory signals that resulted in the expansion and proliferation of a large number of T-cells with migratory capabilities.

Besides their highly therapeutic anti-tumor potential, cell-free vaccines based on MBs have multiple advantages over classical DC vaccines. Due to the smaller size of DC-iMBs compared to DCs, much higher yields of cell-free vaccine can be generated from the same starting quantity of progenitor cells. In this study, we estimated that one activated MoDC generated more than 1000 DC-iMBs, each mimicking an independent mature DC. Such feature can appear extremely useful to turn ineligible patients for conventional DC vaccines into eligible patients for DC-iMB vaccines. In addition to higher yields, cell-free vaccines could enable on-demand vaccine production with reduced cost and manufacturing time and thus may facilitate a greater access to immunotherapy [59]. In contrast to the current mainstream MB made by extrusion or sonication methods, we used a microfluidic platform using a pressure-based disruption and proteolipidic reconstitution process. Microfluidics allow uniform particle sizes and facilitate higher loading of therapeutics, thus improving batch-to-batch reproducibility. This process is also clinically scalable and allows for bubble shell enrichment with any peptide or protein of interest. It makes possible and easy to generate a vaccine which expresses a standardized amount of cytokine/interleukin or immunostimulatory adjuvants by enrichment of bubble shell at the time of microfluidic processing to further potentate T-cell priming. In addition, MB-based vaccines could be stored for longer than 6 months without loss of immunotherapeutic activity. Besides, US contrast agents are uniquely suited for local vaccine delivery. Specifically, focused US pulses can be applied to mechanically push MBs toward the endothelial lining of interest to rupture it. Such targeted sonoporation method has been tested in patients to force the local extravasation of anti-cancer therapeutics and could allow for a precise MB-mediated vaccine delivery to the spleen [17, 60].

To evaluate the effect and efficacy of immunotherapeutics, animals bearing human tumors and human immune systems are required to achieve clinically reliable results for translational applications. NSG mice lack T and B lymphocytes and present with reduced DC and macrophage functions with no complement hemolytic activity nor functional NK cells. Therefore, the humanized immune system of NSG mouse model has been extensively investigated as a preclinical bridge in multiple research areas [61]. As a main advantage, humanized NSG mice re-create the repertoire diversity of human T cells, B cells, and other immune cells enabling investigations on how the human immune system functions in a wide range of diseases. Such models support the co-engraftment of multiple human tissues like primary tumors while retaining their natural architecture. Nevertheless, this model impaired the immunotherapy evaluation period to typically 4–8 weeks because of the short lifespan of PBMCs, and the generation of lethal xGVHD with the onset correlating directly with the levels of human PBMCs used for engraftment [62]. Moreover, the lack of organized lymphoid structures (i.e.*,* germinal centers) and immune cell supply from the bone marrow may limit the development of a robust immune response [63].

## Conclusion

Fundamental in biology, the cell membrane possesses a wide range of functions, including immune escape, long blood circulation time, specific molecular recognition, and cell targeting. Biomimetic MBs could be formulated using numerous naturally derived cell membranes endowing them with unique properties. Recently, we exploited the homotypic recognition of a TNBC cell line to their parent cancer cells to synthesize a tumor targeted-US contrast agent for diagnostic molecular imaging purposes. Our probe demonstrated increased extravasation and retention in a TNBC mouse model compared to the targeted one by CEUS imaging and was further validated by immunofluorescence analysis, allowing a more rapid, safe, and accurate breast lesions screening [24].Altogether, the early findings presented in the present study support the potential of DC-iMBs as a novel immunotherapeutic cell-free anti-cancer vaccine. Looking toward clinical translation, DC-iMB vaccines may be derived from a patient’s own tumor and immune cells, which would ensure that the most appropriate set of antigens are used to train the immune system. Ultimately, continued development along the lines of personalized biomimetic vaccines may significantly change the current clinical landscape of cancer therapy by overcoming tumor heterogeneity.

## Supplementary Information

**Additional file 1: Supplementary scheme 1.** Biomimetic MB generation. **Figure S1.** Flow cytometry analysis of sorted CD14-positive cell population from hPBMCs of 3 healthy donors. Cell surface markers CD45, CD14, and CD11c were assessed pre- and post- CD14-positive cell enrichment and represented as histograms. **Figure S2.** Western blot analysis of different cellular fractions from MDA-MB-231 cells isolated by a differential centrifugation method for various cellular markers. The samples were probed using antibodies against GAPDH, Histone 3, Cytochrome C, and N-Cadherin. **Figure S3.** Evaluation of monocyte differentiation, maturation, and activation into mature moDCs by flow cytometry using a panel of immune markers. **Figure S4.** Size (top) and surface potential (bottom) characterization of MBs and DC-iMBs. **Figure S5.** Blank and plasma membrane impregnated MB morphology by SEM (25.13 K magnification). Scale bar = 1 μm. **Figure S6.** FACS analysis of NSG mouse peripheral blood after 2 doses of hPBMCs for hCD45 (top) and mCD45 (bottom) on day 12. **Figure S7.** *Ex vivo* analysis of therapeutic treatment on tumor growth. **Figure S8.** Histological analysis on major organs and tumors of animals from different treatment groups. **Figure S9.** FACS analysis of MHC II, CD83, and CD4/CD8 cell-positive populations in the spleen, lymph nodes, blood, thymus, tumor and lungs from various treatment groups. **Table S1.** Characteristics of the different treatment groups. **Table S2.** Slope values of tumor volume and tumor volume change pre- and post- different treatments.

## Data Availability

Data are available on reasonable request. All data relevant to the study are included in the article or uploaded as supplementary information.

## Abbreviations

ACT: Adoptive T-cell therapy
APC: Antigen presenting cell;
APC: Allophycocyanin
BC: Breast cancer
BCA: Bicinchoninic acid assay
CCM: Cancer cell membrane
CD: Cluster of differentiation
CEUS: Contrast enhanced ultrasound
CTL: Cytotoxic T lymphocyte
DC: Dendritic cell
DCm: Dendritic cell membrane
DLS: Dynamic light scattering
DMEM: Dulbecco’s Modified Eagle Medium
ER: Estrogen receptor
FACS: Fluorescence-activated cell sorting
FBS: Fetal bovine serum
FDA: U.S. Food and Drug Administration
FITC: Fluorescein isothiocyanate
GAPDH: Glyceraldehyde-3-Phosphate Dehydrogenase
GM-CSF: Granulocyte macrophage colony stimulating factor
HER2: Human epidermal growth factor 2-receptor
hPBMC: Human peripheral blood mononuclear cell
ICI: Immune checkpoint inhibitor
IL: Interleukin
iMB: Immunotheranostic microbubble
iMODC: Immature monocyte derived dendritic cell
LN: Lymph node
MACS: Magnetic-activated cell sorting
MB: Microbubble
MHC: Major histocompatibility complex
NK: Natural killer
ORR: Objective response rate
PBS: Phosphate-buffered saline
PE: Phycoerythrin
PL: Phospholipid
SCID: Severe combined immunodeficient
SEM: Scanning electron microscopy
TAA: Tumor-associated antigen
TCR: T-cell receptor
TIL: Tumor infiltrating lymphocyte
TLR: Toll-like receptor
TNBC: Triple negative breast cancer
TSA: Tumor-specific antigen
US: Ultrasound
WCL: Whole cell lysate
xGVHD: Graft versus host disease
