# Correction: Immunotheranostic microbubbles (iMBs) - a modular platform for dendritic cell vaccine delivery applied to breast cancer immunotherapy

**DOI:** 10.1186/s13046-022-02577-x

**Published:** 2022-12-24

**Authors:** Natacha Jugniot, Jeremy J. Dahl, Ramasamy Paulmurugan

**Affiliations:** 1 Department of Radiology, Molecular Imaging Program at Stanford, Canary Center for Cancer Early Detection, Stanford University, Palo Alto, CA USA; 2 Molecular Imaging Program at Stanford (MIPS), Canary Center for Cancer Early Detection at Stanford, Stanford University School of Medicine, 3155 Porter Drive, Palo Alto, CA 94304 USA

**Correction:** ***J ExpClin Cancer Res*** **41, 299 (2022)**

**https://doi.org/10.1186/s13046-022-02501-3**

Following publication of the original article [1], author noticed an incoherence in the SEM image of targeted NBs shown in Figure S5. The correct Figures S5 has been updated.

This correction does not change the result, interpretation, and conclusions of the study. The original article has been corrected.

## Supplementary Information

**Additional file 1: Supplementary Figure S5.** Blank and plasma membrane impregnated MB morphology by SEM (25.13 K magnification). Scale bar = 1 μm.
