# Supplementary material for: Immunotheranostic microbubbles (iMBs) - a modular platform for dendritic cell vaccine delivery applied to breast cancer immunotherapy
Source: J Exp Clin Cancer Res. 2022 Oct 12;41:299. doi: 10.1186/s13046-022-02501-3 (PMC9555090; doi:10.1186/s13046-022-02501-3)
Supplement: Supplementary file 1 — Additional file 1: Supplementary scheme 1. Biomimetic MB generation. Figure S1. Flow cytometry analysis of sorted CD14-positive cell population from hPBMCs of 3 healthy donors. Cell surface markers CD45, CD14, and CD11c were assessed pre- and post- CD14-positive cell enrichment and represented as histograms. Figure S2. Western blot analysis of different cellular fractions from MDA-MB-231 cells isolated by a differential centrifugation method for various cellular markers. The samples were probed using antibodies against GAPDH, Histone 3, Cytochrome C, and N-Cadherin. Figure S3. Evaluation of monocyte differentiation, maturation, and activation into mature moDCs by flow cytometry using a panel of immune markers. Figure S4. Size (top) and surface potential (bottom) characterization of MBs and DC-iMBs. Figure S5. Blank and plasma membrane impregnated MB morphology by SEM (25.13 K magnification). Scale bar = 1 μm. Figure S6. FACS analysis of NSG mouse peripheral blood after 2 doses of hPBMCs for hCD45 (top) and mCD45 (bottom) on day 12. Figure S7. Ex vivo analysis of therapeutic treatment on tumor growth. Figure S8. Histological analysis on major organs and tumors of animals from different treatment groups. Figure S9. FACS analysis of MHC II, CD83, and CD4/CD8 cell-positive populations in the spleen, lymph nodes, blood, thymus, tumor and lungs from various treatment groups. Table S1. Characteristics of the different treatment groups. Table S2. Slope values of tumor volume and tumor volume change pre- and post- different treatments. [file 13046_2022_2501_MOESM1_ESM.pdf]

<u>Supplementary Information</u>

Immunotheranostic Microbubbles (*i*MBs) - A Modular Platform for Dendritic Cell Vaccine Delivery Applied to Breast Cancer Immunotherapy

Natacha Jugniot, Ph.D.[1] Jeremy J. Dahl[1], and Ramasamy Paulmurugan, Ph.D.[1*]

[1]Department of Radiology, Molecular Imaging Program at Stanford, Canary Center for Cancer Early Detection, Stanford University, Palo Alto, CA, USA.

Supplementary Scheme and Figures

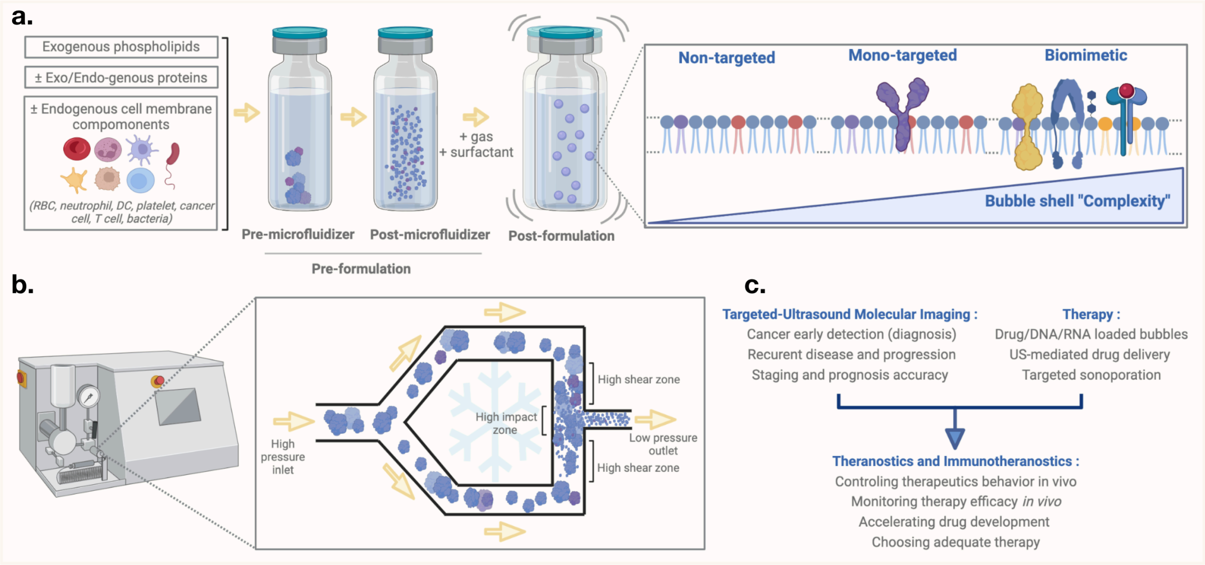

Supplementary scheme 1. Biomimetic MB generation. (a) MBs can be synthesized with increasing shell "complexity" starting with commercial phospholipids (*i.e.,* non-targeted MBs), enriched with synthetic or natural proteins (*i.e.,*"mono"-targeted MBs), and/or formulated with cell membrane fragments from various sources (*i.e.,* biomimetic MBs). Using a microfluidizer device, the fragments can be solubilized with homogenized size. Finally, upon the addition of gas and surfactant, vigorous mechanical vibration allows the formation of monodispersed MBs. **(b)** Exposure of liquids containing macromolecules under high shear rates inside a microfluidizer "Y"-type interaction chamber; self-assembled particles can be reclaimed at the outlet. **(c)** The potential clinical applications of biomimetic MBs are broad and likely to impact many oncological patient's outcomes in various ways. Applications include targeted-molecular US imaging of tumors, targeted-drug delivery, and therapeutic and theranostic development.

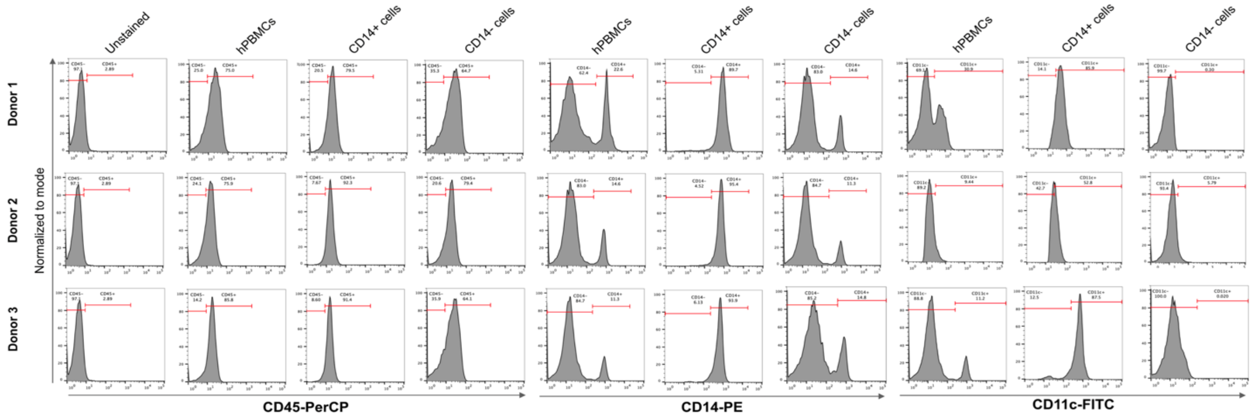

Figure S1. Flow cytometry analysis of sorted CD14-positive cell population from hPBMCs of 3 healthy donors. Cell surface markers CD45, CD14, and CD11c were assessed pre- and post-CD14-positive cell enrichment and represented as histograms.

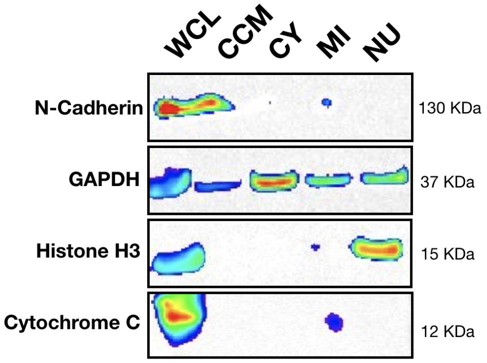

Figure S2. Western blot analysis of different cellular fractions from MDA-MB-231 cells isolated by a differential centrifugation method for various cellular markers. The samples were probed using antibodies against GAPDH, Histone 3, Cytochrome C, and N-Cadherin.

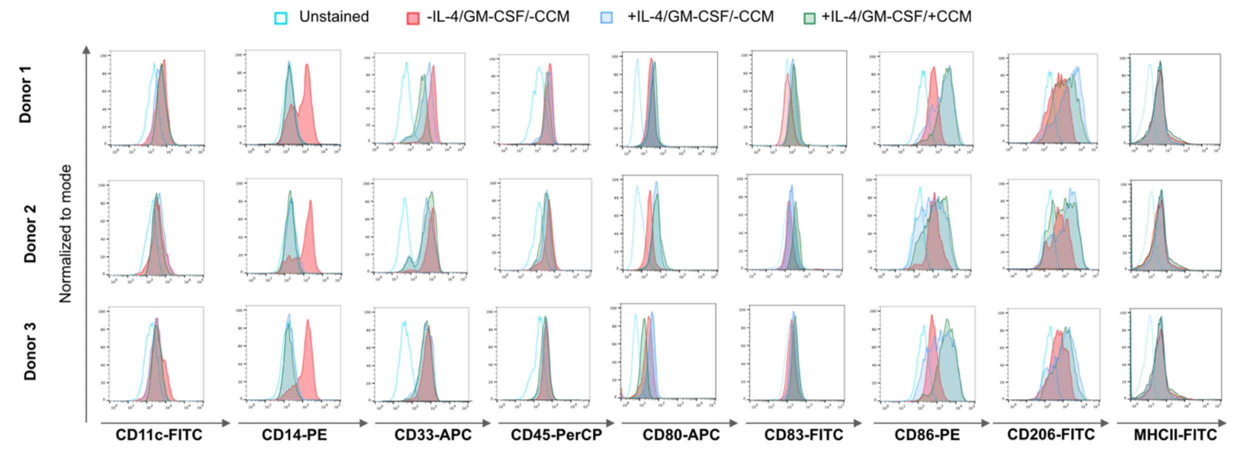

Figure S3. Evaluation of monocyte differentiation, maturation, and activation into mature moDCs by flow cytometry using a panel of immune markers.

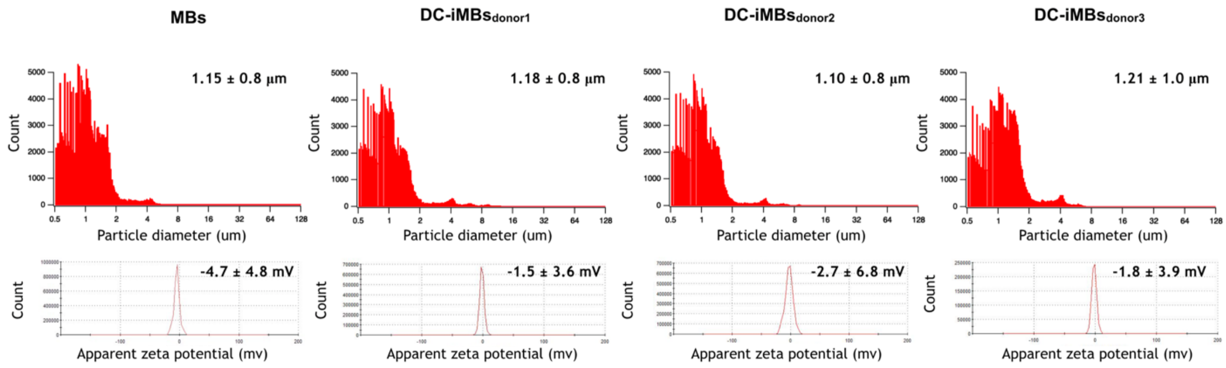

Figure S4. Size (top) and surface potential (bottom) characterization of MBs and DC-iMBs.

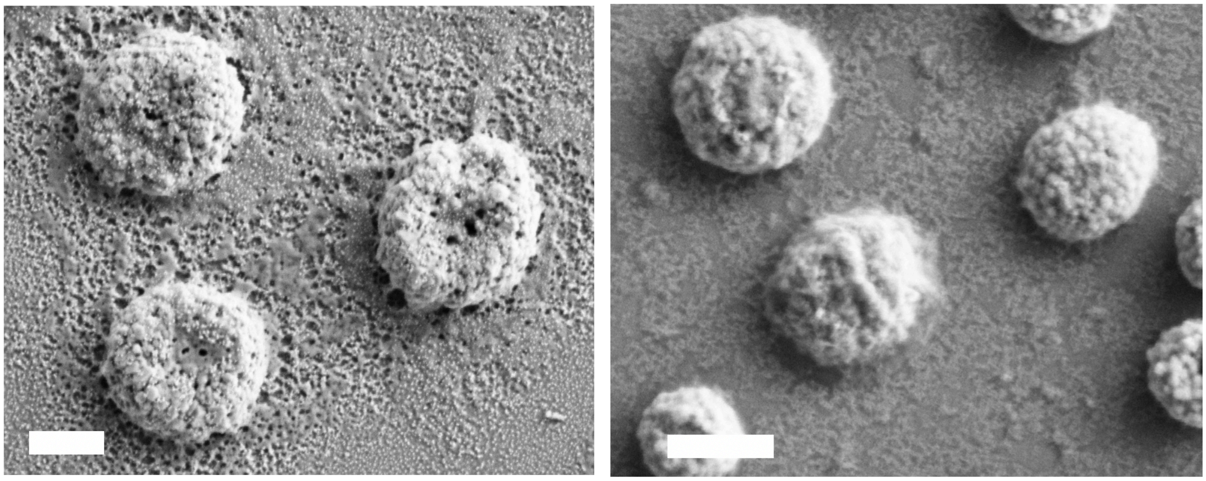

Figure S5. Blank (left) and plasma membrane impregnated (right) MB morphology by SEM (25.13 K magnification). Scale bar = 1 μm.

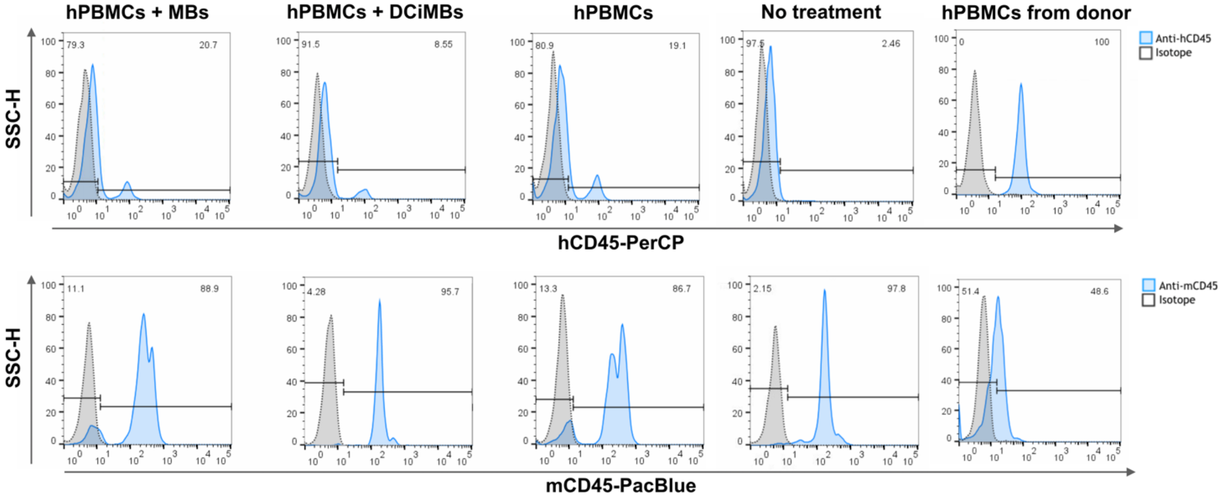

Figure S6. FACS analysis of NSG mouse peripheral blood after 2 doses of hPBMCs for hCD45 (top) and mCD45 (bottom) on day 12.

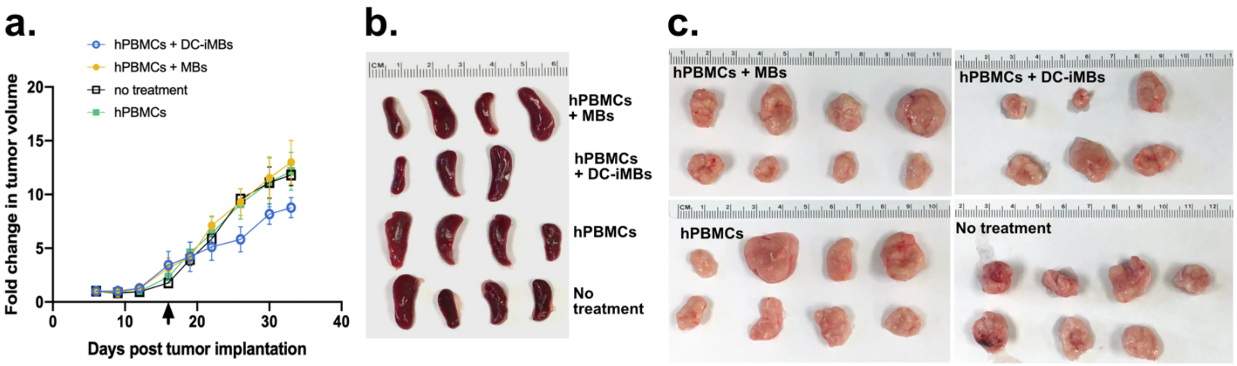

Figure S7. ***Ex vivo* analysis of therapeutic treatment on tumor growth. (a)** Relative change in tumor volume over time. Black arrows indicate the starting date of therapeutic treatments; **(b)** *Ex vivo* photographs of spleens from different treatment groups at the end of the study; and **(c)** *Ex vivo* photographs of tumors from different treatment groups at the end of the study.

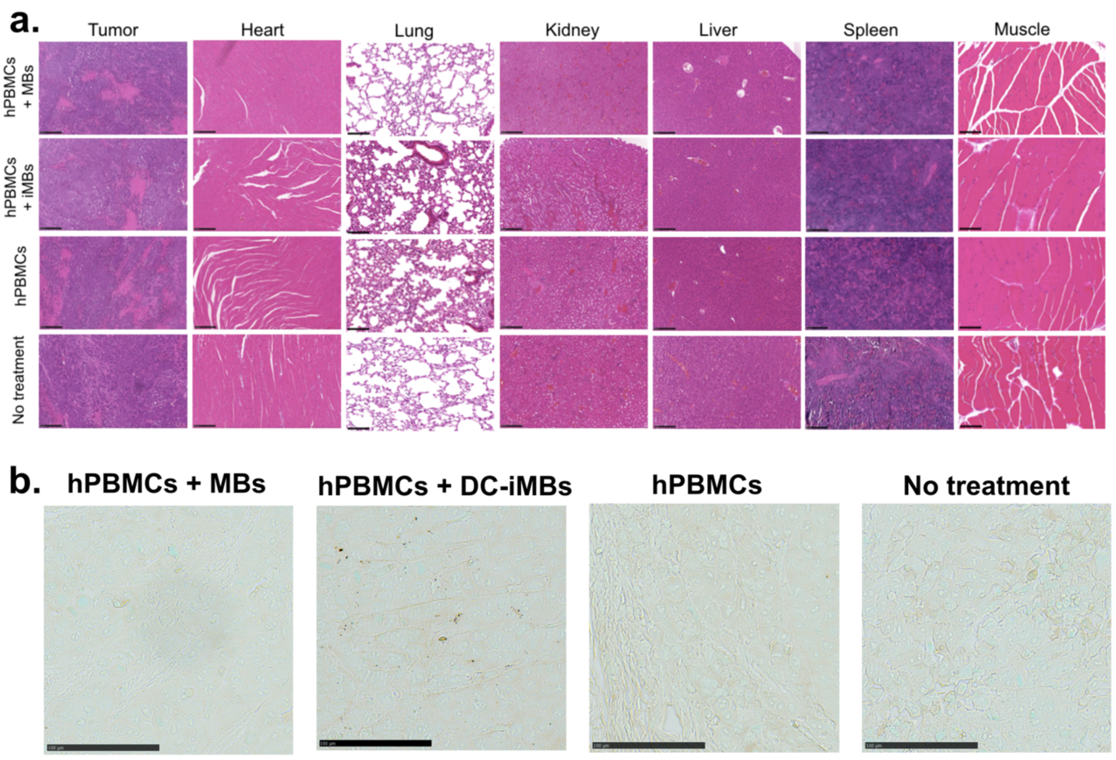

Figure S8. **Histological analysis on major organs and tumors of animals from different treatment groups. (a)** H&E-stained histologic sections of mouse tissues from different treatment groups (heart, lung, tumor, kidney, liver, spleen, lymph node, and thymus treated by MB, DC-iMBs, or PBS respectively). Scale bar = 100 µm. **(b)** Representative images of apoptosis using tumor sections across the different conditions of treatment. Scale bar = 100 µm.

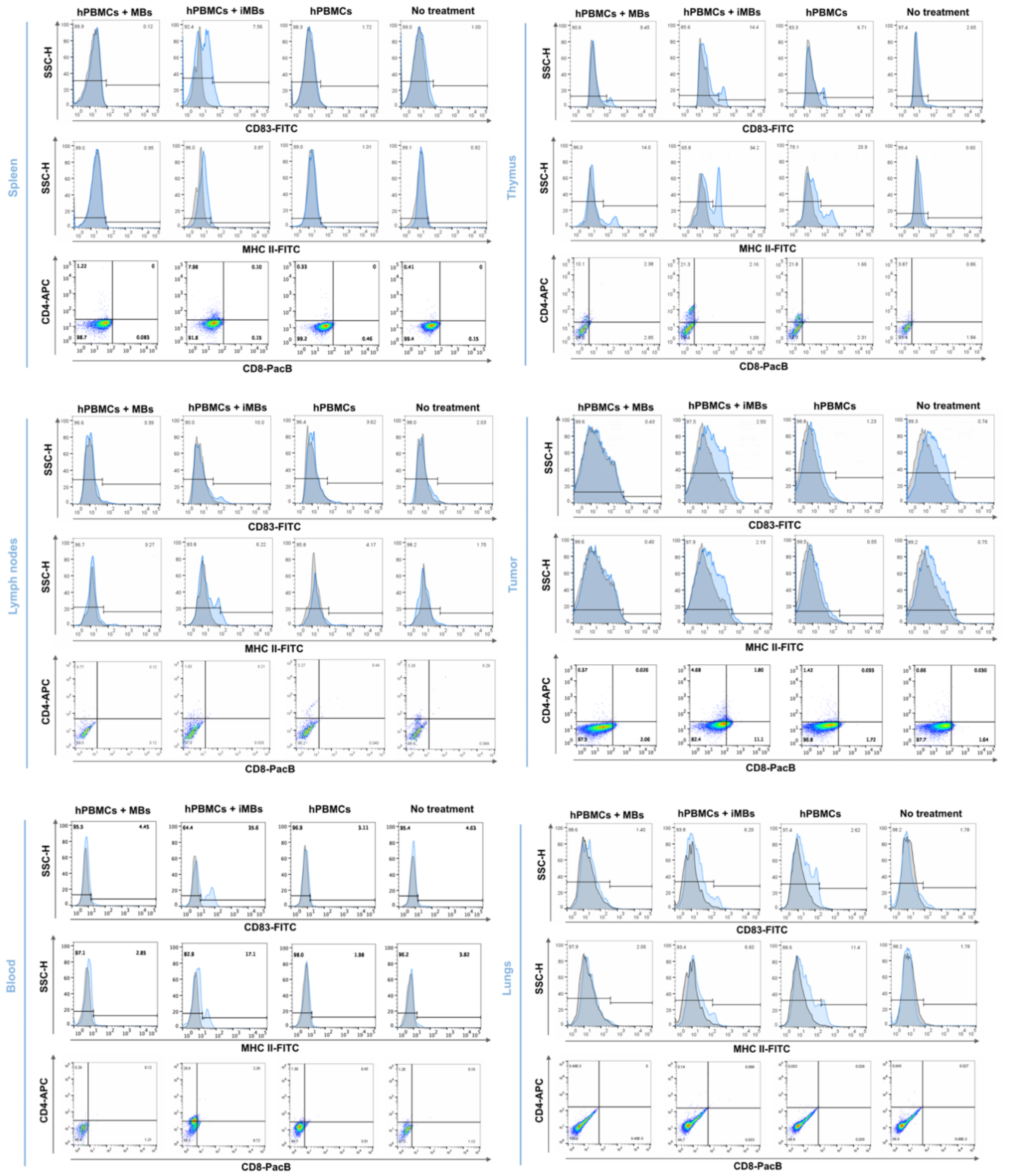

Figure S9. FACS analysis of MHC II, CD83, and CD4/CD8 cell-positive populations in the spleen, lymph nodes, blood, thymus, tumor and lungs from various treatment groups.

Supplementary Table

Group	n	T-cell engraftment	Anti-TNCB vaccination		Description
			Treatment/Dose	Composition	
1	4	hPBMC (10^7cells, days 5&7, 200 µL, iv)	MB (10^7 particles, day 16, 100 µL, iv)	Lipids+gas+surfactant	Control MB in human immune system
2	4	hPBMC (10^7cells, days 5&7, 200 µL, iv)	DC-iMB (10^7 particles, day 16, 100 µL, iv)	Mature DC membrane+lipids +gas+surfactant	Immunotherapeutic MB in human immune system
3	4	hPBMC (10^7cells, days 5&7, 200 µL, iv)	PBS (control, day 16, 100 µL, iv))	-	Negative control (in human immune system)
4	4	PBS (control, days 5&7, 200 µL, iv)	PBS (control, day 16, 100 µL, iv)	-	Negative control (no human immune system)

Table S1. Characteristics of the different treatment groups.

Cohort	Fold change slop of tumor volume pre-treatment (D0-D16)	Fold change slop of tumor volume post-treatment (D16-D33)
hPBMC + MBs	+ 0.26 ± 0.05	+ 0.61 ± 0.04
hPBMC + iMBs	+ 0.27 ± 0.05	+ 0.33 ± 0.03 ***
hPBMC	+ 0.25 ± 0.08	+ 0.53 ± 0.03
No treatment	+ 0.26 ± 0.08	+ 0.55 ± 0.03

Table S2. Slope values of tumor volume and tumor volume change pre- and post- different treatments.
